# Supplementary material for: GCalignR: An R package for aligning gas-chromatography data for ecological and evolutionary studies
Source: PLoS One. 2018 Jun 7;13(6):e0198311. doi: 10.1371/journal.pone.0198311 (PMC5991698; doi:10.1371/journal.pone.0198311)
Supplement: S1 File — (DOCX) [file pone.0198311.s001.docx]

**S1. Summary of published algorithms implemented in publicly available software for aligning peaks in LC-MS, GC-MS and GC-FID datasets.**

Blank entries indicate that the information was not accessible from the manuscript. See the main manuscript for details on the bibliographic search.

| Algorithm | Designed for | Input data | Used Dimensions | Code available | Environment | Peak scoring | Reference | Strictly pairwise | Sensitive to intensity variation | Visual tools | Warping |
| --- | --- | --- | --- | --- | --- | --- | --- | --- | --- | --- | --- |
| GCalignR | GC-FID | Peak list | RT | Yes | R | Yes | Automatic,  User | No | No | Yes | No |
| amsrpm[1] | LC-MS | Peak list | RT, I, m/z  RT, I | Yes | R | No | User | Yes | Yes | No | Yes |
| BAM[2] | LC-MS | Raw MS | RT | Yes | Matlab |  | Consensus derived from internal standards |  |  | No | Yes |
| Bylund *et al.[3]* | LC-MS | BPC | I, m/z | No | Matlab | No | User | Yes | Yes | No | Yes |
| ChromA[4] | GC-MS | Raw MS | I, m/z | No | Java | No | User | Yes | Yes | Yes | Yes |
| CPM[5] | LC-MS | Raw MS | RT, I, m/z | Yes | Matlab |  |  |  | Yes | No | Yes |
| eRah[6] | GC-MS | Raw MS | RT, m/z | Yes | R | Yes | None | No |  |  |  |
| GCALIGNER 1.0 [7] | GC-FID | Peak list | RT | No | Java | Yes | None | No | No | No | No |
| IDEAL-Q[8] | LC-MS/MS | Raw MS | RT, I, m/z | No |  |  | User |  | Yes | Yes | Yes |
| LWBMatch[9] | LC-MS | Raw MS | RT, m/z | Yes | C++ |  | User,  Automatic | Partly |  | No | Yes |
| MultiAlign[10] | LC-MS | Raw MS | RT, I, m/z | No | C/C++ | Requires database |  |  | Yes | Yes | Yes |
| MassUntangler[11] | LC-MS | Peak list | RT, I, m/z | Yes | Python 2.6.2 |  |  | Yes | Yes | No | No |
| MetAlign[12] | GC-MS LC-MS | Raw MS | RT, I, m/z | No |  |  |  |  | Yes | No | Yes |
| msInspect/AMT[13] | LC-MS/MS | Raw MS | RT, I, m/z | Yes | Java, R | Yes, using AMT database | AMT database |  | Yes | No | No |
| MZmine 2[14] | GC-MS  LC-MS | Raw MS | RT, I, m/z |  | Java | Yes |  |  | Yes | Yes | Yes |
|  |  |  |  |  |  |  |  |  |  |  |  |
|  |  |  |  |  |  |  |  |  |  |  |  |
| Msalign[15] | LC-MS/MS + LC-MS | Raw MS | I, m/z | Yes | C |  |  |  | Yes | No | Yes |
| Peakmatch[16] |  | TICC | RT | Yes | Matlab | No | User | Yes | Yes | No | No |
| PEPPeR[17] | LC-MS |  | RT, m/z | Yes | Perl | Yes | User | Yes | No | No | Yes |
| PETAL[18] | LC-MS | Raw MS | RT, m/z | Yes | R |  |  | No |  | No | No |
| Podwojski *et al.[19]* | LC-MS | MS peak list | RT, I, m/z | Yes | R |  |  |  | Yes | No | Yes |
| ptw[20,21] | LC-MS | Raw MS  Peak list | I, RT | Yes | R | No | User | Yes | Yes | No | Yes |
| SIMA[22] | LC-MS | Peak list | RT, m/z | Yes | C++ |  | None | No |  | No | Yes |
| SpecArray[23] | LC-MS | Raw MS | RT, m/z | No | C |  |  |  |  |  | Yes |
| SuperHirn[24] | LC-MS | Raw MS | RT, m/z | Yes | C++ |  | None | No |  |  |  |
| Warp2D[25,26] | LC-MS | Peak List | RT, I, m/z | No | Java | No | User | Yes | Yes | No | Yes |
| XCMS[27] | LC-MS | Raw MS | RT, I, m/z | Yes | R | Yes |  | Yes | Yes | Yes | Yes |

Abbreviations: BPC = Base peak chromatogram [28], GC-FID = Gas chromatography coupled to a flame ionization detector, GC-MS = Gas chromatography mass-spectrometry, I = Peak intensity, LC-MS = Liquid chromatography mass-spectrometry, m/z = mass-over-charge, RT = retention time, TICC =Chromatogram created by plotting the total ion current in a series of mass spectra recorded as a function of retention time [28]

**References**

1. Kirchner M, Saussen B, Steen H, Steen JAJ, Hamprecht FA. amsrpm: Robust point matching for retention time alignment of LC/MS data with R. JOURNAL OF STATISTICAL SOFTWARE. 2007; 18.

2. Tsai T-H, Tadesse MG, Di Poto C, Pannell LK, Mechref Y, Wang Y, et al. Multi-profile Bayesian alignment model for LC-MS data analysis with integration of internal standards. BIOINFORMATICS. 2013; 29: 2774–2780.

3. Bylund D, Danielsson R, Malmquist G, Markides KE. Chromatographic alignment by warping and dynamic programming as a pre-processing tool for PARAFAC modelling of liquid chromatography–mass spectrometry data. JOURNAL OF CHROMATOGRAPHY A. 2002; 961: 237–244.

4. Hoffmann N, Stoye J. ChromA: signal-based retention time alignment for chromatography-mass spectrometry data. BIOINFORMATICS. 2009; 25: 2080–2081. doi: 10.1093/bioinformatics/btp343.

5. Listgarten J, Neal RM, Roweis ST, Wong P, Emili A. Difference detection in LC-MS data for protein biomarker discovery. BIOINFORMATICS. 2007; 23: e198-e204.

6. Domingo-Almenara X, Brezmes J, Vinaixa M, Samino S, Ramirez N, Ramon-Krauel M, et al. eRah: A Computational Tool Integrating Spectral Deconvolution and Alignment with Quantification and Identification of Metabolites in GC/MS-Based Metabolomics. ANALYTICAL CHEMISTRY. 2016; 88: 9821–9829. doi: 10.1021/acs.analchem.6b02927.

7. Dellicour S, Lecocq T. GCALIGNER 1.0: An alignment program to compute a multiple sample comparison data matrix from large eco‐chemical datasets obtained by GC. JOURNAL OF SEPARATION SCIENCE. 2013; 36: 3206–3209.

8. Tsou C-C, Tsai C-F, Tsui Y-H, Sudhir P-R, Wang Y-T, Chen Y-J, et al. IDEAL-Q, an automated tool for label-free quantitation analysis using an efficient peptide alignment approach and spectral data validation. MOLECULAR & CELLULAR PROTEOMICS. 2010; 9: 131–144.

9. Wang J, Lam H. Graph-based peak alignment algorithms for multiple liquid chromatography-mass spectrometry datasets. BIOINFORMATICS. 2013; 29: 2469–2476. doi: 10.1093/bioinformatics/btt435.

10. LaMarche BL, Crowell KL, Jaitly N, Petyuk VA, Shah AR, Polpitiya AD, et al. MultiAlign: a multiple LC-MS analysis tool for targeted omics analysis. BMC BIOINFORMATICS. 2013; 14: 49. doi: 10.1186/1471-2105-14-49.

11. Ballardini R, Benevento M, Arrigoni G, Pattini L, Roda A. MassUntangler: A novel alignment tool for label-free liquid chromatography–mass spectrometry proteomic data. JOURNAL OF CHROMATOGRAPHY A. 2011; 1218: 8859–8868.

12. Lommen A. MetAlign: interface-driven, versatile metabolomics tool for hyphenated full-scan mass spectrometry data preprocessing. ANALYTICAL CHEMISTRY. 2009; 81: 3079–3086.

13. May D, Fitzgibbon M, Liu Y, Holzman T, Eng J, Kemp CJ, et al. A platform for accurate mass and time analyses of mass spectrometry data. JOURNAL OF PROTEOME RESEARCH. 2007; 6: 2685–2694.

14. Pluskal T, Castillo S, Villar-Briones A, Orešič M. MZmine 2: modular framework for processing, visualizing, and analyzing mass spectrometry-based molecular profile data. BMC BIOINFORMATICS. 2010; 11: 395.

15. Palmblad M, Mills DJ, Bindschedler LV, Cramer R. Chromatographic alignment of LC-MS and LC-MS/MS datasets by genetic algorithm feature extraction. JOURNAL OF THE AMERICAN SOCIETY FOR MASS SPECTROMETRY. 2007; 18: 1835–1843.

16. Johnson KJ, Wright BW, Jarman KH, Synovec RE. High-speed peak matching algorithm for retention time alignment of gas chromatographic data for chemometric analysis. JOURNAL OF CHROMATOGRAPHY A. 2003; 996: 141–155. doi: 10.1016/S0021-9673(03)00616-2.

17. Jaffe JD, Mani, Leptos KC, Church GM, Gillette MA, Carr SA. PEPPeR, a platform for experimental proteomic pattern recognition. MOLECULAR & CELLULAR PROTEOMICS. 2006; 5: 1927–1941.

18. Wang P, Tang H, Fitzgibbon MP, McIntosh M, Coram M, Zhang H, et al. A statistical method for chromatographic alignment of LC-MS data. Biostatistics. 2006; 8: 357–367.

19. Podwojski K, Fritsch A, Chamrad DC, Paul W, Sitek B, Stühler K, et al. Retention time alignment algorithms for LC/MS data must consider non-linear shifts. BIOINFORMATICS. 2009; 25: 758–764.

20. Bloemberg TG, Gerretzen J, Wouters HJP, Gloerich J, van Dael M, Wessels HJ, et al. Improved parametric time warping for proteomics. CHEMOMETRICS AND INTELLIGENT LABORATORY SYSTEMS. 2010; 104: 65–74.

21. Wehrens R, Bloemberg TG, Eilers PHC. Fast parametric time warping of peak lists. BIOINFORMATICS. 2015; 31: 3063–3065.

22. Voss B, Hanselmann M, Renard BY, Lindner MS, Köthe U, Kirchner M, et al. SIMA: simultaneous multiple alignment of LC/MS peak lists. BIOINFORMATICS. 2011; 27: 987–993.

23. Li X-j, Eugene CY, Kemp CJ, Zhang H, Aebersold R. A software suite for the generation and comparison of peptide arrays from sets of data collected by liquid chromatography-mass spectrometry. MOLECULAR & CELLULAR PROTEOMICS. 2005; 4: 1328–1340.

24. Mueller LN, Rinner O, Schmidt A, Letarte S, Bodenmiller B, Brusniak M, et al. SuperHirn–a novel tool for high resolution LC‐MS‐based peptide/protein profiling. PROTEOMICS. 2007; 7: 3470–3480.

25. Suits F, Lepre J, Du P, Bischoff R, Horvatovich P. Two-Dimensional Method for Time Aligning Liquid Chromatography− Mass Spectrometry Data. ANALYTICAL CHEMISTRY. 2008; 80: 3095–3104.

26. Ahmad I, Suits F, Hoekman B, Swertz MA, Byelas H, Dijkstra M, et al. A high-throughput processing service for retention time alignment of complex proteomics and metabolomics LC-MS data. BIOINFORMATICS. 2011; 27: 1176–1178. doi: 10.1093/bioinformatics/btr094.

27. Smith CA, Want EJ, O’Maille G, Abagyan R, Siuzdak G. XCMS: Processing mass spectrometry data for metabolite profiling using Nonlinear peak alignment, matching, and identification. ANALYTICAL CHEMISTRY. 2006; 78: 779–787. doi: 10.1021/ac051437y.

28. Murray KK, Boyd RK, Eberlin MN, Langley GJ, Li L, Naito Y. Definitions of terms relating to mass spectrometry (IUPAC Recommendations 2013). Pure and Applied Chemistry. 2013; 85: 43. doi: 10.1351/PAC-REC-06-04-06.
