# Supplementary material for: GCalignR: An R package for aligning gas-chromatography data for ecological and evolutionary studies
Source: PLoS One. 2018 Jun 7;13(6):e0198311. doi: 10.1371/journal.pone.0198311 (PMC5991698; doi:10.1371/journal.pone.0198311)
Supplement: S2 File — (DOCX) [file pone.0198311.s002.docx]

**Supporting Information S5. Literature survey on the prevalence of manual alignment of corresponding peaks**

We conducted a full-text search using Google Scholar for the journals “Animal Behaviour” and “Proceedings of royal society B” using the following keywords:

(Scent OR Odour) AND (GC-FID OR GC-MS OR Chromatography) AND Similarity

The initial list was comprised of 66 studies that were filtered for empirical studies on chemical profiles of animals or plant volatiles that deployed Gas Chromatography in the form of either GC-FID, GC-MS or both methods on which further analyses were based. For all these publications, we scanned the Methods section and the supplementary files where required for details on the processing of the chemical data after the analytical. In no case we found evidence for the use of any published algorithm for the assembly of peak list prior to statistical analyses.

References

1. Akino T, Knapp JJ, Thomas JA, Elmes GW. Chemical mimicry and host specificity in the butterfly Maculinea rebeli, a social parasite of Myrmica ant colonies. Proceedings of the Royal Society of London B: Biological Sciences. 1999; 266: 1419–1426.

2. Ali JG, Tallamy DW. Female spotted cucumber beetles use own cuticular hydrocarbon signature to choose immunocompatible mates. Animal Behaviour. 2010; 80: 9–12.

3. Boulay R, Cerdá X, Simon T, Roldan M, Hefetz A. Intraspecific competition in the ant Camponotus cruentatus: should we expect the ‘dear enemy’effect. Animal Behaviour. 2007; 74: 985–993.

4. Breed MD, Garry MF, Pearce AN, Hibbard BE, BJOSTAD LB, PAGE Jr RE. The role of wax comb in honey bee nestmate recognition. Animal Behaviour. 1995; 50: 489–496.

5. Burgener N, Dehnhard M, Hofer H, East ML. Does anal gland scent signal identity in the spotted hyaena. Animal Behaviour. 2009; 77: 707–715.

6. Cornille A, Underhill JG, Cruaud A, Hossaert-McKey M, Johnson SD, Tolley KA, et al. Floral volatiles, pollinator sharing and diversification in the fig–wasp mutualism: insights from Ficus natalensis, and its two wasp pollinators (South Africa). Proceedings of the Royal Society of London B: Biological Sciences. 2011: rspb20111972.

7. Crawford JC, Boulet M, Drea CM. Smelling wrong: hormonal contraception in lemurs alters critical female odour cues. Proceedings of the Royal Society of London B: Biological Sciences. 2011; 278: 122–130.

8. Cunningham JP, Moore CJ, Zalucki MP, Cribb BW. Insect odour perception: recognition of odour components by flower foraging moths. Proceedings of the Royal Society of London B: Biological Sciences. 2006; 273: 2035–2040.

9. Cuvillier-Hot V, Lenoir A, Crewe R, Malosse C, Peeters C. Fertility signalling and reproductive skew in queenless ants. Animal Behaviour. 2004; 68: 1209–1219.

10. d'Ettorre P, Mondy N, Lenoir A, Errard C. Blending in with the crowd: social parasites integrate into their host colonies using a flexible chemical signature. Proceedings of the Royal Society of London B: Biological Sciences. 2002; 269: 1911–1918.

11. Foitzik S, Sturm H, Pusch K, D'Ettorre P, Heinze J. Nestmate recognition and intraspecific chemical and genetic variation in Temnothorax ants. Animal Behaviour. 2007; 73: 999–1007.

12. Fürst MA, Durey M, Nash DR. Testing the adjustable threshold model for intruder recognition on Myrmica ants in the context of a social parasite. Proceedings of the Royal Society of London B: Biological Sciences. 2011: rspb20110581.

13. Greene LK, Drea CM. Love is in the air: sociality and pair bondedness influence sifaka reproductive signalling. Animal Behaviour. 2014; 88: 147–156.

14. Haberer W, Steiger S, Müller JK. (E)-methylgeranate, a chemical signal of juvenile hormone titre and its role in the partner recognition system of burying beetles. Animal Behaviour. 2010; 79: 17–24.

15. Henneken J, Jones TM, Goodger JQD, Dias DA, Walter A, Elgar MA. Diet influences female signal reliability for male mate choice. Animal Behaviour. 2015; 108: 215–221.

16. Johnson CA, Phelan PL, Herbers JM. Stealth and reproductive dominance in a rare parasitic ant. Animal Behaviour. 2008; 76: 1965–1976.

17. Kölliker M, Chuckalovcak JP, Haynes KF, Brodie ED. Maternal food provisioning in relation to condition-dependent offspring odours in burrower bugs (Sehirus cinctus). Proceedings of the Royal Society of London B: Biological Sciences. 2006; 273: 1523–1528.

18. Kroiss J, Bordon S, Strohm E. Hydrocarbons in the nest material of a solitary digger wasp represent a kairomone for a specialized cuckoo wasp. Animal Behaviour. 2008; 76: 1555–1563.

19. Leclaire S, Merkling T, Raynaud C, Mulard H, Bessière J-M, Lhuillier É, et al. Semiochemical compounds of preen secretion reflect genetic make-up in a seabird species. Proceedings of the Royal Society of London B: Biological Sciences. 2012; 279: 1185–1193.

20. Li J, Wang Z, Tan K, Qu Y, Nieh JC. Giant Asian honeybees use olfactory eavesdropping to detect and avoid ant predators. Animal Behaviour. 2014; 97: 69–76.

21. Lorenzi MC, Cervo R, Bagnères A-G. Facultative social parasites mark host nests with branched hydrocarbons. Animal Behaviour. 2011; 82: 1143–1149.

22. Mitra A, Ramachandran A, Gadagkar R. Nestmate discrimination in the social wasp Ropalidia marginata: chemical cues and chemosensory mechanism. Animal Behaviour. 2014; 88: 113–124.

23. Nunes TM, Nascimento FS, Turatti IC, Lopes NP, Zucchi R. Nestmate recognition in a stingless bee: does the similarity of chemical cues determine guard acceptance. Animal Behaviour. 2008; 75: 1165–1171.

24. Nunes TM, Mateus S, Turatti IC, Morgan ED, Zucchi R. Nestmate recognition in the stingless bee Frieseomelitta varia (Hymenoptera, Apidae, Meliponini): sources of chemical signals. Animal Behaviour. 2011; 81: 463–467.

25. Pareja M, Mohib A, Birkett MA, Dufour S, Glinwood RT. Multivariate statistics coupled to generalized linear models reveal complex use of chemical cues by a parasitoid. Animal Behaviour. 2009; 77: 901–909.

26. Ponzio C, Cascone P, Cusumano A, Weldegergis BT, Fatouros NE, Guerrieri E, et al. Volatile-mediated foraging behaviour of three parasitoid species under conditions of dual insect herbivore attack. Animal Behaviour. 2016; 111: 197–206.

27. Quezada-Euán JJG, Ramírez J, Eltz T, Pokorny T, Medina R, Monsreal R. Does sensory deception matter in eusocial obligate food robber systems? A study of Lestrimelitta and stingless bee hosts. Animal Behaviour. 2013; 85: 817–823.

28. Rasmussen LE, Wittemyer G. Chemosignalling of musth by individual wild African elephants (Loxodonta africana): implications for conservation and management. Proceedings of the Royal Society of London B: Biological Sciences. 2002; 269: 853–860.

29. Reichle C, Aguilar I, Ayasse M, Twele R, Francke W, Jarau S. Learnt information in species-specific ‘trail pheromone’communication in stingless bees. Animal Behaviour. 2013; 85: 225–232.

30. Safi K, Kerth G. Secretions of the interaural gland contain information about individuality and colony membership in the Bechstein's bat. Animal Behaviour. 2003; 65: 363–369.

31. Setchell JM, Vaglio S, Abbott KM, Moggi-Cecchi J, Boscaro F, Pieraccini G, et al. Odour signals major histocompatibility complex genotype in an Old World monkey. Proceedings of the Royal Society of London B: Biological Sciences. 2010: rspb20100571.

32. Shuttleworth A, Johnson SD. The missing stink: sulphur compounds can mediate a shift between fly and wasp pollination systems. Proceedings of the Royal Society of London B: Biological Sciences. 2010: rspb20100491.

33. Signorotti L, Jaisson P, D'Ettorre P. Larval memory affects adult nest-mate recognition in the ant Aphaenogaster senilis. Proceedings of the Royal Society of London B: Biological Sciences. 2014; 281: 20132579.

34. Steinmetz I, Schmolz E, Ruther J. Cuticular lipids as trail pheromone in a social wasp. Proceedings of the Royal Society of London B: Biological Sciences. 2003; 270: 385–391.

35. Stökl J, Brodmann J, Dafni A, Ayasse M, Hansson BS. Smells like aphids: orchid flowers mimic aphid alarm pheromones to attract hoverflies for pollination. Proceedings of the Royal Society of London B: Biological Sciences. 2011; 278: 1216–1222.

36. Tasin M, Knudsen GK, Pertot I. Smelling a diseased host: grapevine moth responses to healthy and fungus-infected grapes. Animal Behaviour. 2012; 83: 555–562.

37. Vásquez GM, Silverman J. Intraspecific aggression and colony fusion in the Argentine ant. Animal Behaviour. 2008; 75: 583–593.

38. Wong JWY, Meunier J, Lucas C, Kölliker M. Paternal signature in kin recognition cues of a social insect: concealed in juveniles, revealed in adults. Proceedings of the Royal Society of London B: Biological Sciences. 2014; 281: 20141236.
