# Supplementary material for: GCalignR: An R package for aligning gas-chromatography data for ecological and evolutionary studies
Source: PLoS One. 2018 Jun 7;13(6):e0198311. doi: 10.1371/journal.pone.0198311 (PMC5991698; doi:10.1371/journal.pone.0198311)
Supplement: S3 File — All analysis steps are provided in an Rmarkdown document file. (PDF) [file pone.0198311.s003.pdf]

R-code for ‘GCalignR: An R package for aligning Gas-Chromatography data’

Meinolf Ottensmann, Martin A. Stoffel, Hazel J. Nichols, Joseph I. Hoffman

This document provides all the `R code` used for our paper. Both the Rmarkdown file and the data can be downloaded from the accompanying GitHub repository on (https://github.com/mottensmann/GCalignR_Supplementary) as a zip archive containing all the files. For computational reasons we provide the results of time consuming steps in addition to the raw data on GitHub.

Prerequisites

Most functions that are required to run the code presented within this document are part of our R package `GCalignR` that needs to be installed first.

- Installing `GCalignR`.

```
# install GCalignR
install.packages("GCalignR")
```

Additional functions are provided in form of `R` scripts that are available in the sub-directory `R`. In order to run the code you need to have a sub-directory called `data` that contains the raw data files. Furthermore, the are some packages that are not distributed with base R that have to be installed from CRAN.

- Install `devtools` , `ggplot2`, `plot3D` and `vegan` if these packages are not available.

```
# install devtools
if (!("devtools" %in% rownames(installed.packages()))) {
    install.packages("devtools")
} else if (packageVersion("devtools") < 1.6) {
    install.packages("devtools")
}
# install ggplot2
if (!"ggplot2" %in% rownames(install.packages())) {
    install.packages("ggplot2")
}
# install gridExtra
if (!"gridExtra" %in% rownames(install.packages())) {
    install.packages("gridExtra")
}
# install plot3D
if (!"plot3D" %in% rownames(installed.packages())) {
    install.packages("plot3D")
}
# install ptw
if (!"ptw" %in% rownames(installed.packages())) {
    install.packages("ptw")
}
# install vegan
if (!"vegan" %in% rownames(installed.packages())) {
    install.packages("vegan")
}
```

- Load packages and source custom functions.

```
library(GCalignR)
library(ggplot2)
library(gridExtra)
library(plot3D)
library(vegan)
library(ptw)
# small function to test parameters in align_chromatograms
source("R/optimal_params.R")
# calculates errors by matching aligned data to a table of
# known substances
source("R/error_rate.R")
# custom function for simulations based on chromatograms
source("R/ChromaSimFunctions.R")
## functions for plotting
source("R/NMDS-Functions.R")
## converts aligned data from GCALIGNER format to GCalignR
source("R/convert2GCalign.R")
## convert data to be used with ptw
source("R/Convert2ptw.R")
## calculation of descriptive stats for a data frame
source("R/summary_stats.R")
```

Demonstration of the workflow

In this section we provide an extended workflow of the dataset presented in the manuscript as well as resulting output to function calls. First, we submit the path to the text file containing the input chemical dataset to the the variable `fpath` and ensure that the text file is formatted properly by running the function `check_input` that would return warnings in case of formatting issues:

```
library(GCalignR)  # loads the package
fpath <- system.file(dir = "extdata", file = "peak_data.txt",
    package = "GCalignR")  # path to peak_data.txt
check_input(fpath)  # checks the data
#> All checks passed!
```

The resulting output of the call to `check_input` "All checks passed!" indicates that the input file meets the data format of `GCalignR`. Next, we align the dataset using the core function `align_chromatograms`. By specifying the negative control samples (i.e. `blanks`) and setting the parameter `delete_single_peak` to `TRUE` we include to optional filtering steps that are executed after the alignment was conducted. Alternatively, these optional steps can be included in the workflow independent of the alignment with `align_chromatograms` by using the functions `remove_blanks` and `remove_singletons` respectively. See the corresponding helpfiles and the vignettes for details on both.

```
# align the chemical data
aligned_peak_data <- align_chromatograms(data = peak_data, rt_col_name = "time",
    max_diff_peak2mean = 0.01, min_diff_peak2peak = 0.08, max_linear_shift = 0.05,
    delete_single_peak = TRUE, blanks = c("C2", "C3"))
```

Afterwards, a summary of the alignment process can be retrieved using the printing method, which summarises the function call including defaults that were not altered by the user. This provides all of the relevant information to retrace every step of the alignment procedure.

```r
print(aligned_peak_data)
#> Summary of Peak Alignment running align_chromatograms
#> Input: peak_data
#> Start:  2017-07-19 16:31:47  Finished:  2017-07-19 17:30:47
#>
#> Call:
#>   GCalignR::align_chromatograms(data=peak_data, rt_col_name=time,
#>   max_linear_shift=0.05, max_diff_peak2mean=0.02, min_diff_peak2peak=0.08,
#>   blanks=(C2, C3), delete_single_peak=T, sep=\t, rt_cutoff_low=NULL,
#>   rt_cutoff_high=NULL, reference=NULL)
#>
#> Summary of scored substances:
#>    total   blanks singular retained
#>      494      171       45      278
#>
#> In total 494 substances were identified among all samples. 171 substances were
#>   present in blanks. The corresponding peaks as well as the blanks were removed
#>   from the data. 45 substances were present in just one single sample and were
#>   removed. 278 substances are retained after all filtering steps.
#>
#> Sample overview:
#>   The following 84 samples were aligned to the reference 'P20':
#>   M2, M3, M4, M5, M6, M7, M8, M9, M10, M12, M14, M15, M16, M17, M18, M19, M20,
#>   M21, M23, M24, M25, M26, M27, M28, M29, M30, M31, M33, M35, M36, M37, M38, M39,
#>   M40, M41, M43, M44, M45, M46, M47, M48, P2, P3, P4, P5, P6, P7, P8, P9, P10,
#>   P12, P14, P15, P16, P17, P18, P19, P20, P21, P23, P24, P25, P26, P27, P28, P29,
#>   P30, P31, P33, P35, P36, P37, P38, P39, P40, P41, P43, P44, P45, P46, P47, P48
#>
#> For further details type:
#>   'gc_heatmap(aligned_peak_data)' to retrieve heatmaps
#>   'plot(aligned_peak_data)' to retrieve further diagnostic plots
```

As alignment quality may vary with the parameter values selected by the user, the plot function can be used to output four diagnostic plots. These allow the user to explore how the parameter values affect the resulting alignment and can help flag issues with the raw data.

```r
plot(aligned_peak_data)  # Figure 1
```

Additionally, the alignment results can be visualised using heat maps with the function `gc_heatmap`.

```r
gc_heatmap(aligned_peak_data)
```

Peak normalisation and downstream analyses

In order to account for differences in the total concentration of samples, we provide an additional function `norm_peaks` that can be used to normalise peak abundances.

```r
scent <- norm_peaks(data = aligned_peak_data, rt_col_name = "time",
    conc_col_name = "area", out = "data.frame")
```

Downstream analyses

The output of GCalignR is compatible with other functionalities in R, thereby providing a seamless transition between packages. For instance, multivariate analyses can be conduced using the package vegan (Oksanen

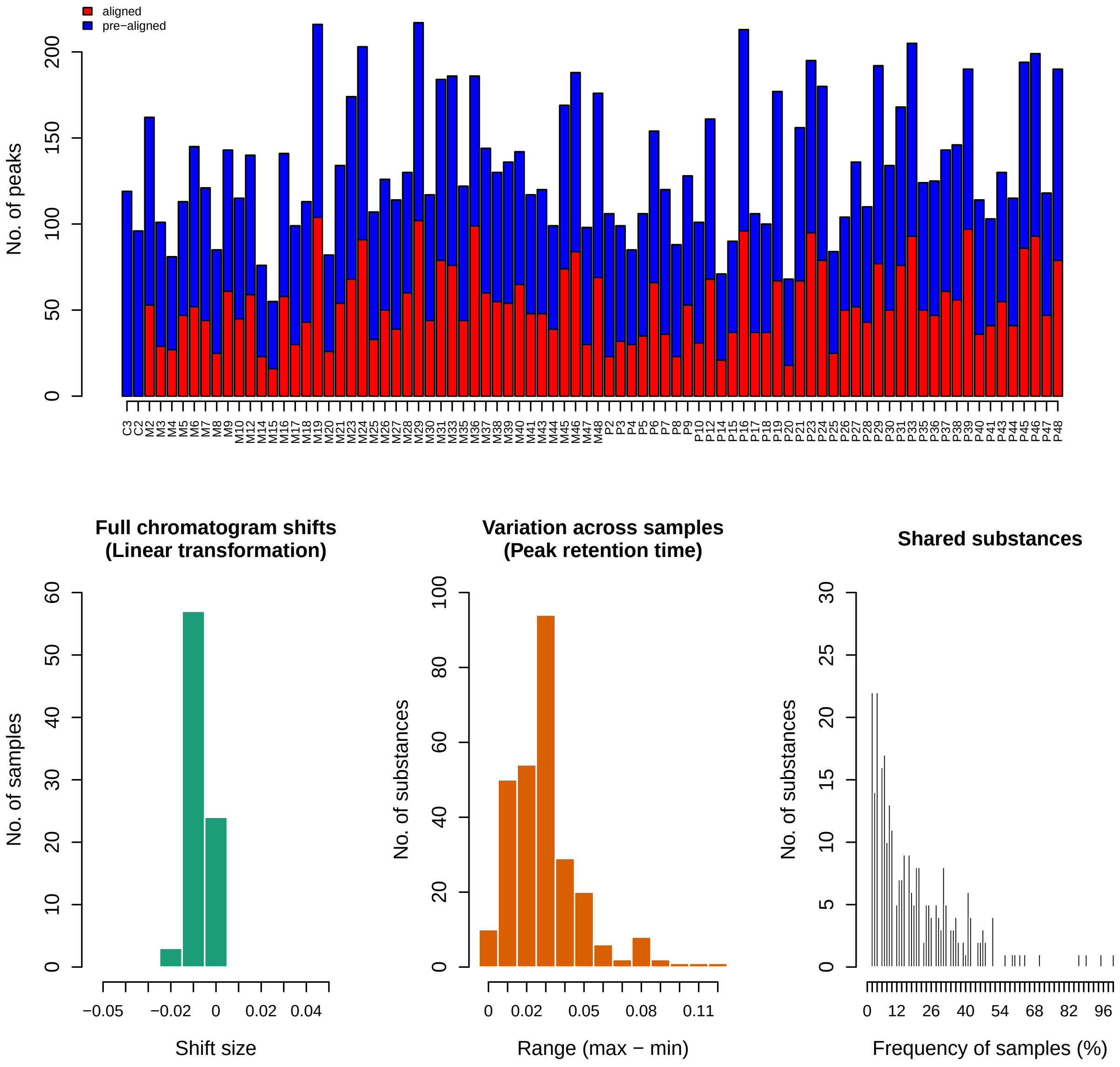

Figure 1: Diagnostic plots summarising the alignment of the Antarctic fur seal chemical dataset.

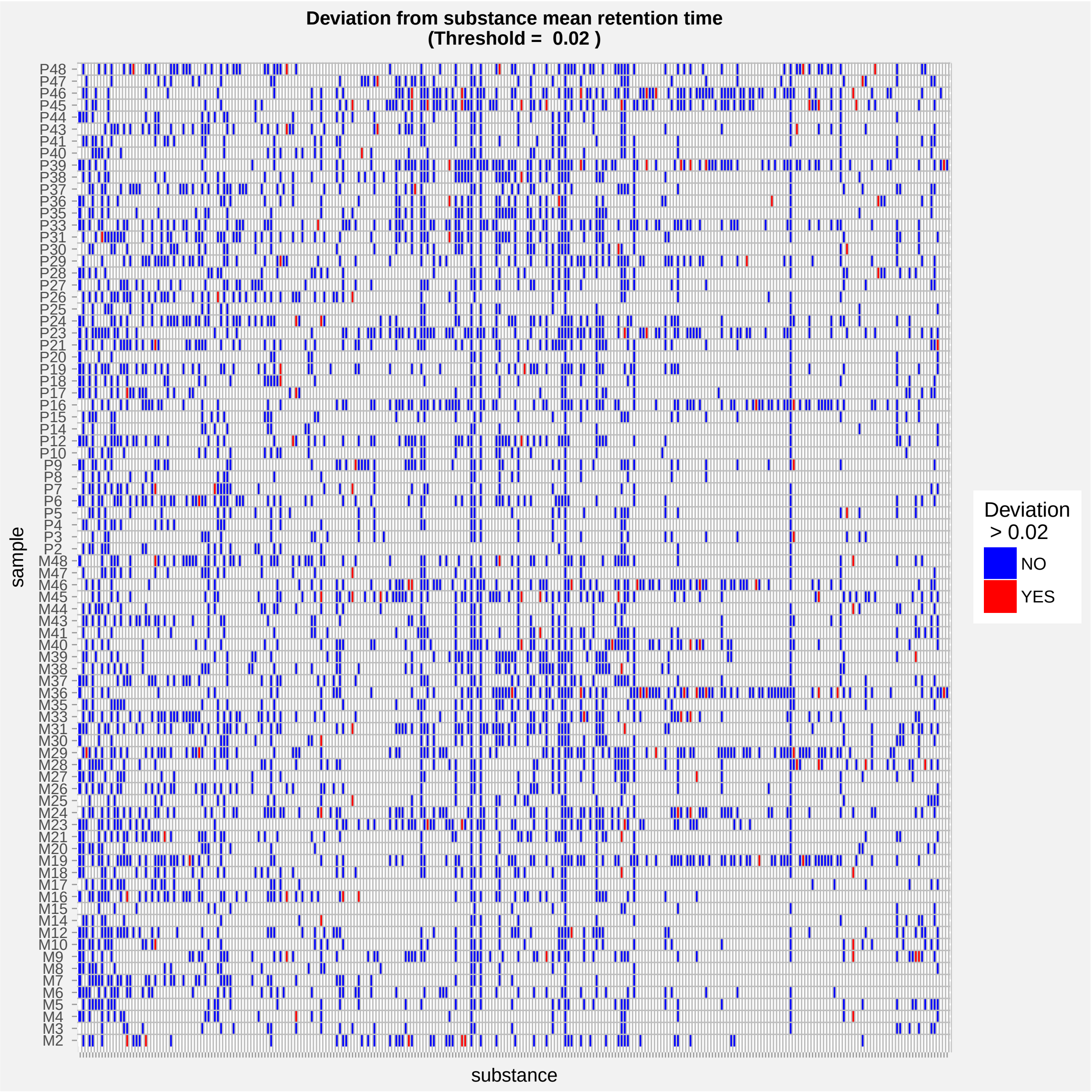

Figure 2: Heatmaps allow to inspect the distribution of substances across samples as well as the variability of their retention times.

et al. 2016). To visualise patterns of chemical similarity within the fur seal dataset in relation to breeding colony membership, we implemented non-metric-multidimensional scaling (NMDS) based on a Bray-Curtis dissimilarity matrix and visualised the outcome using ggplot2 (Wickham 2009).

```
scent <- scent[match(row.names(peak_factors), row.names(scent)),
    ]  # sort data
scent <- log(scent + 1)  # log + 1 transformation
```

```
scent_nmds <- vegan::metaMDS(comm = scent, distance = "bray")  # NMDS
scent_nmds <- as.data.frame(scent_nmds[["points"]])  # extract points
scent_nmds <- cbind(scent_nmds, colony = peak_factors[["colony"]])  # add factors
```

```
# Figure 3
ggplot(data = scent_nmds, aes(MDS1, MDS2, color = colony)) +
    geom_point(size = 2.5) + theme_void() + scale_color_manual(values = c("blue",
    "red")) + theme(panel.background = element_rect(colour = "black",
    size = 1.25, fill = NA), aspect.ratio = 1, legend.position = "none")
```

Comparison with GCALIGNER

To facilitate comparison of `GCalignR` and `GCALIGNER`, we downloaded raw data on cephalic labial gland secretions from three bumblebee species (Dellicour and Lecocq 2013} that is available as supporting information Table S_1 and prepared input files for `GCalignR`. Each of these datasets included data on both known and unknown substances, the former being defined as those substances that were identified with respect to the NIST database using GC-MS. These substances were extracted from Table S_4 and allow to match peaks in the aligned chemical GC-FID dataset to the reference and calculate error rates by:

$$\text{Error} = \left[\frac{\text{Number of misaligned retention times}}{\text{Total number of retention times}}\right] \tag{1}$$

First, we align each dataset with `GCalignR` using the default parameters `max_linear_shift = 0.02`, `max_diff_peak2mean = 0.01` and `min_diff_peak2peak = 0.08`.

```
bfla_GCalignR_default <- align_chromatograms("data/bfla.txt",
    rt_col_name = "RT")
save(bfla_GCalignR_default, file = "data/bfla_GCalignR_default.RData",
    compress = T)
bbim_GCalignR_default <- align_chromatograms("data/bbim.txt",
    rt_col_name = "RT")
save(bbim_GCalignR_default, file = "data/bbim_GCalignR_default.RData",
    compress = T)
beph_GCalignR_default <- align_chromatograms("data/beph.txt",
    rt_col_name = "RT")
save(beph_GCalignR_default, file = "data/beph_GCalignR_default.RData",
    compress = T)
```

Then by retrieving diagnostic plots (functions `plot` and `gc_heatmap`) we inspect alignment results and fine-tune parameters. For computational reasons, we provide the results of the code chunk above that can be loaded into the workspace with:

```
load("data/bfla_GCalignR_default.RData")
load("data/bbim_GCalignR_default.RData")
load("data/beph_GCalignR_default.RData")
```

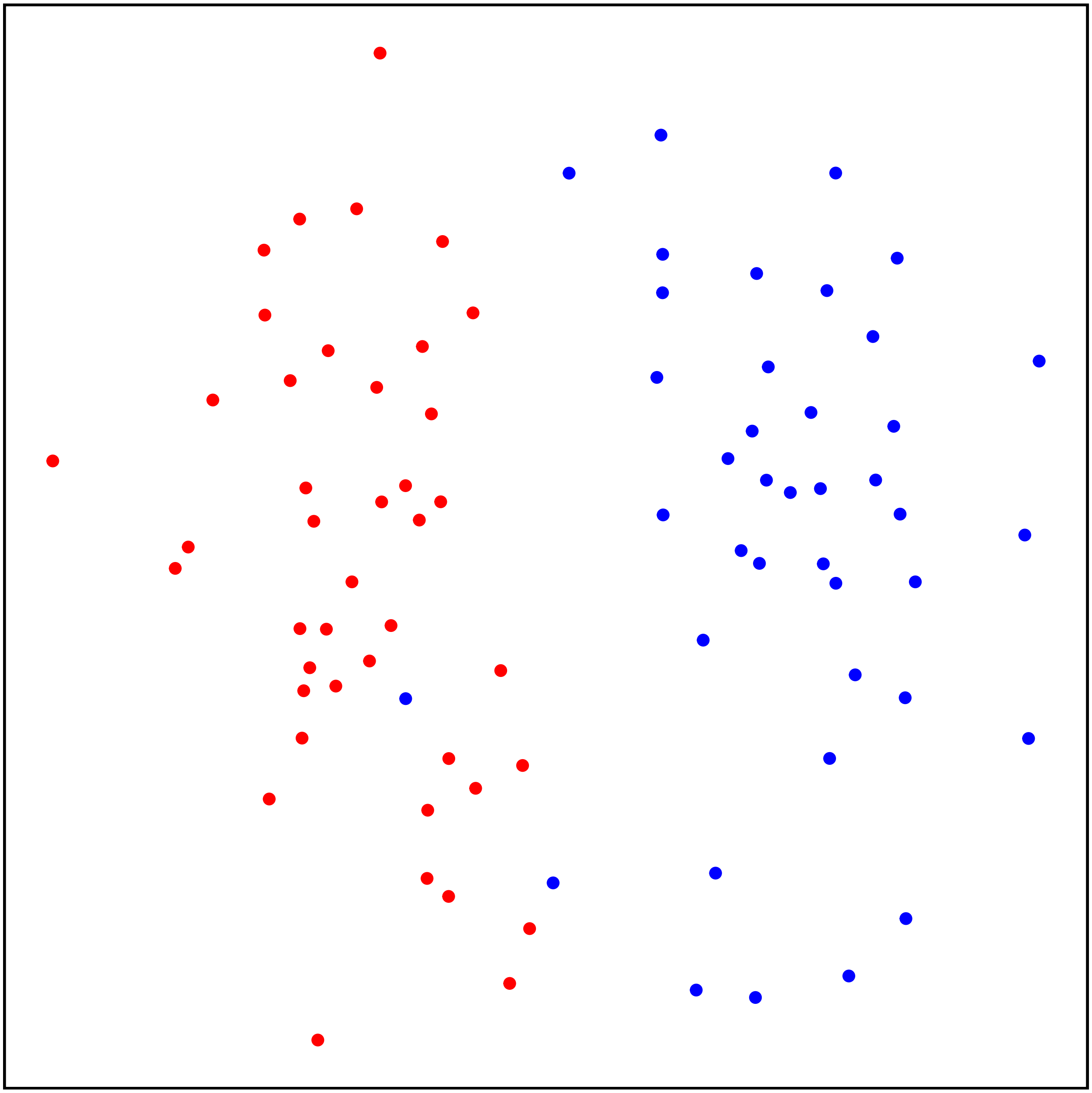

Figure 3: Two-dimensional nonmetric multidimensional scaling plot of chemical data from 41 Antarctic fur seal mother–offspring pairs. Bray–Curtis similarity values were calculated from standardized and log(x+1) transformed abundance data. Individuals from each of the two colonies are shown in blue and red respectively

For all three datasets, only little systematic shifts in retention times of putatively homologous peaks are present in the datasets, as can be seen by the distribution of linear shifts that were applied during the first step of the alignment procedure with `GCalignR`. Consequently, there is no need to increase the window size by adjusting the parameter `max_linear_shift`.

```
par(mfrow = c(1, 3))
plot(bfla_GCalignR_default, which_plot = "shifts", main = "B. flavifrons")
plot(bbim_GCalignR_default, which_plot = "shifts", main = "B. bimaculatus")
plot(beph_GCalignR_default, which_plot = "shifts", main = "B. ephippiatus")
```

However, heat maps show patterns in all three datasetes that indicate that putatively homologous rows exist that can be merged by increasing the value of the parameter `min_diff_peak2peak`.

```
p1 <- gc_heatmap(bfla_GCalignR_default, main = "Alignment with default parameters",
    label = "y", show_legend = F)

p2 <- gc_heatmap(bfla_GCalignR_default, main = "A subset of substances",
    label = "xy", substance_subset = 15:30, show_legend = F)

grid.arrange(p1, p2, nrow = 1, ncol = 2)
```

For example, in the aligned chemical dataset of *B. flavifrons* such a pattern is shown by two rows are that are annotated with '20.23' and '20.71' referring to the mean retention time of the corresponding rows (Figure 5, right panel). In order to allow merging such rows, we fine-tune the alignment by setting `min_diff_peak2peak = 0.48` and run the alignment again.

```
bfla_GCalignR_optimised <- align_chromatograms("data/bfla.txt",
    rt_col_name = "RT", max_diff_peak2mean = 0.01, min_diff_peak2peak = 0.48)
save(bfla_GCalignR_optimised, file = "data/bfla_GCalignR_optimised.RData")

bbim_GCalignR_optimised <- align_chromatograms("data/bbim.txt",
    rt_col_name = "RT", max_diff_peak2mean = 0.01, min_diff_peak2peak = 0.48)
save(bbim_GCalignR_optimised, file = "data/bbim_GCalignR_optimised.RData")

beph_GCalignR_optimised <- align_chromatograms("data/beph.txt",
    rt_col_name = "RT", max_diff_peak2mean = 0.01, min_diff_peak2peak = 0.48)
save(beph_GCalignR_optimised, file = "data/beph_GCalignR_optimised.RData")
```

```
load("data/bfla_GCalignR_optimised.RData")
load("data/bbim_GCalignR_optimised.RData")
load("data/beph_GCalignR_optimised.RData")
```

Heat maps of the fine-tuned alignment show that the two rows corresponding to putatively homologous substances are now merged within the row annotated by '20.59'

```
p1 <- gc_heatmap(bfla_GCalignR_optimised, main = "Alignment with fine-tuned parameters",
    label = "y", show_legend = F)

p2 <- gc_heatmap(bfla_GCalignR_optimised, main = "A subset of substances)",
    label = "xy", substance_subset = 15:30, show_legend = F)

grid.arrange(p1, p2, nrow = 1, ncol = 2)
```

Dellicour and Lecocq (2013) published optimised alignments using the software `GCALIGNER`. The results are available in for all three datasets in the supporting information Table S_2. The format combines all input variables (RT = retention time, RA = relative peak area and $Area$ = peak area) aligned similarly to `GCalignR` rowise. The function `convert2GCalign` converts each dataset into the format of `GCalignR` in which a matrix

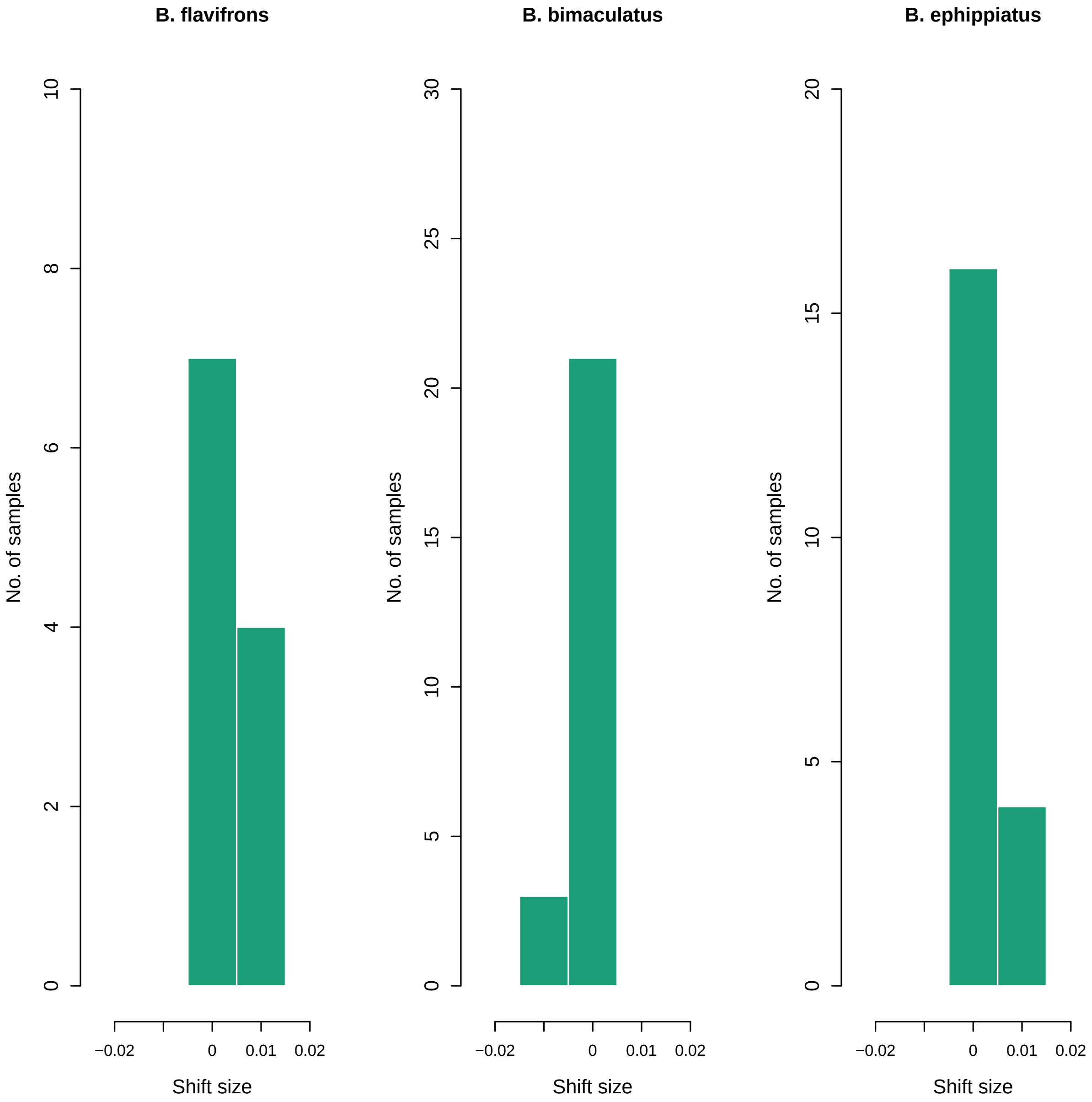

Figure 4: Distribution of applied linear shifts during the first step of the alignment procedure for three bumblebee datasets show overall modest systematic shifts in retention times among samples.

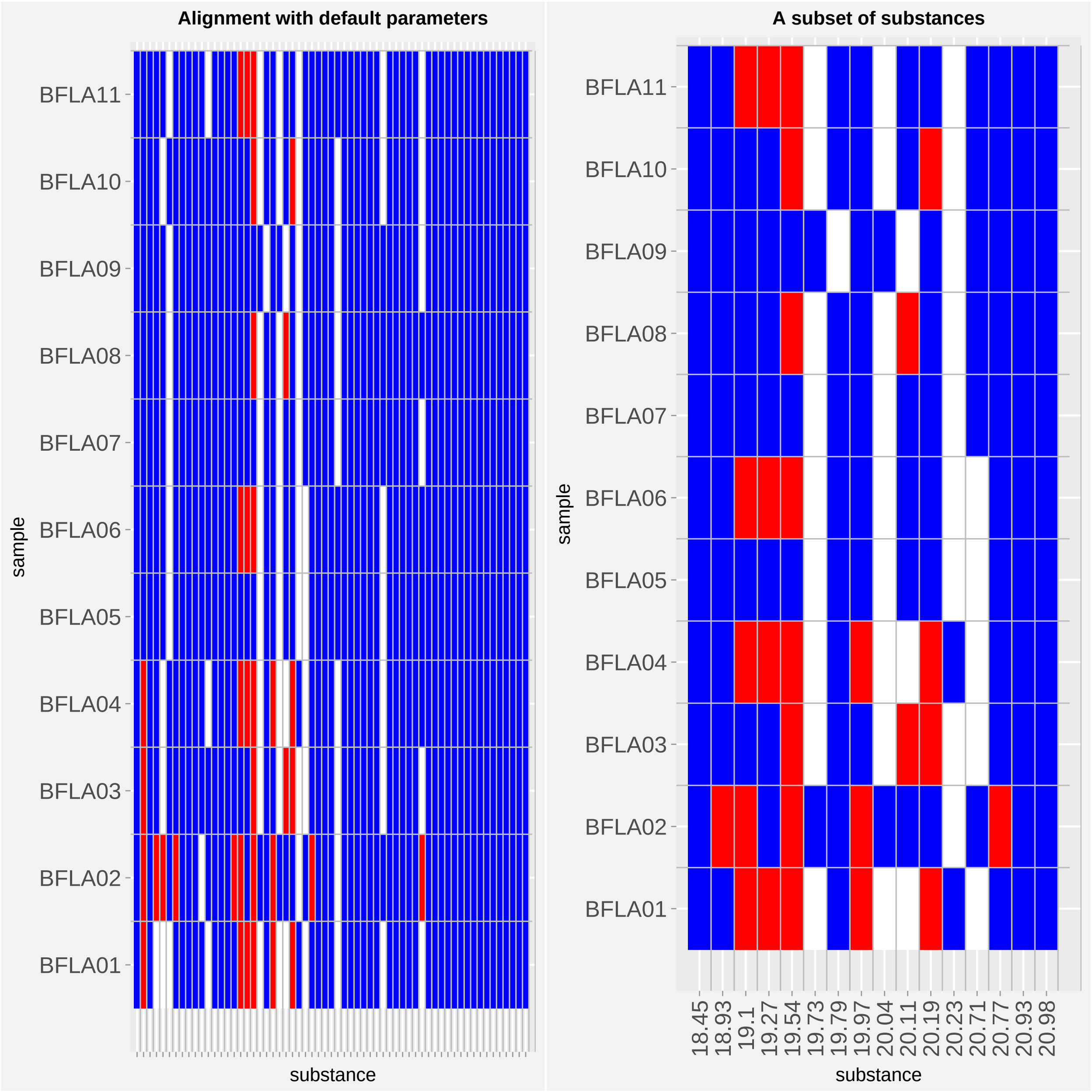

Figure 5: Heatmaps showing the distribution of substances in the aligned dataset using default parameter values. The left panel shows the complete dataset and the right panels shows an excerpt.

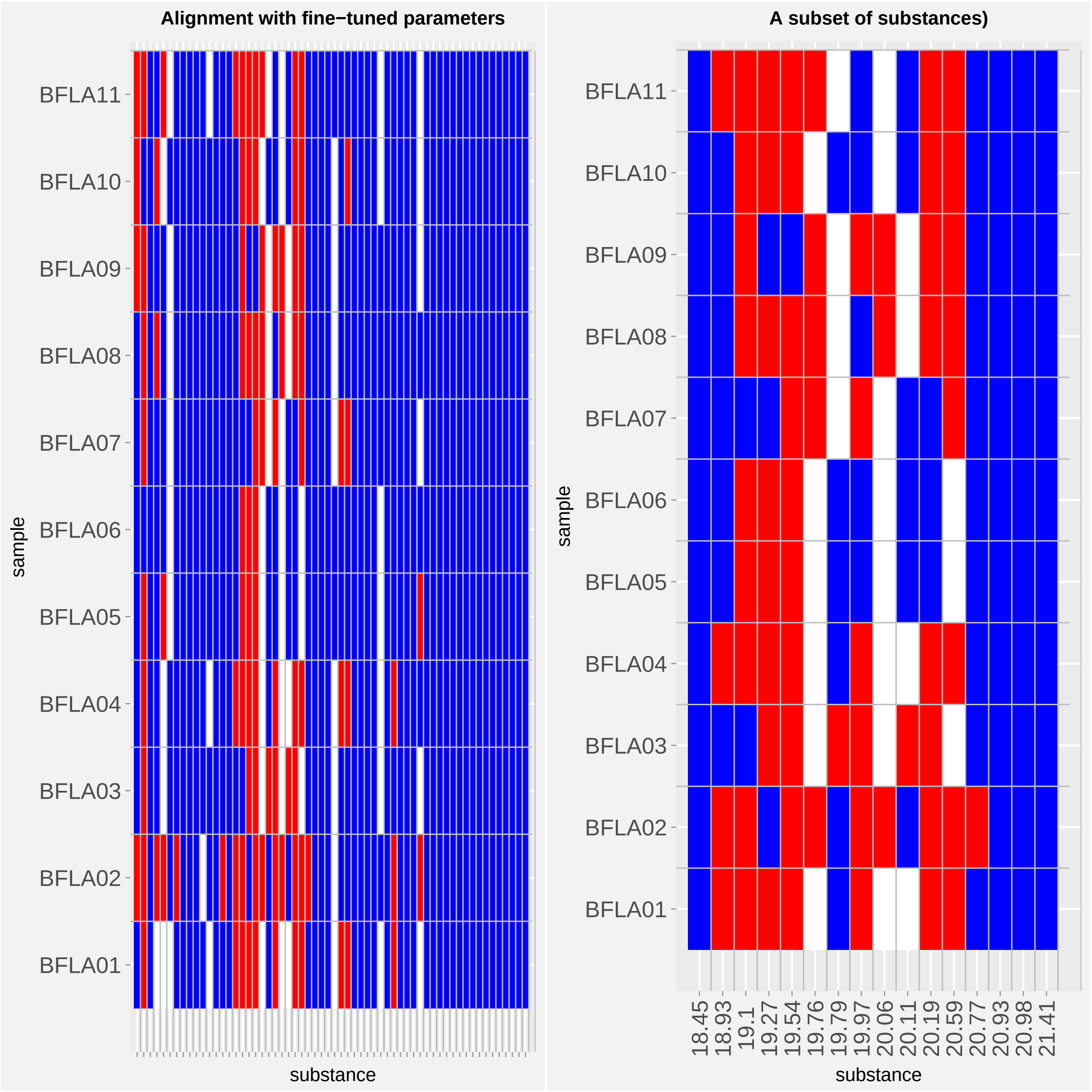

Figure 6: Heatmaps showing the distribution of substances in the aligned dataset. The left panel shows the complete dataset and the right panels shows an excerpt.

containing aligned data for every variable is created.

```
bbim_GCALIGNER <- convert2GCalign(path = "data/Table_S2.xls",
    var_names = c("RT", "Area", "RA"), sheet = 1)

beph_GCALIGNER <- convert2GCalign(path = "data/Table_S2.xls",
    var_names = c("RT", "Area", "RA"), sheet = 2)

bfla_GCALIGNER <- convert2GCalign(path = "data/Table_S2.xls",
    var_names = c("RT", "Area", "RA"), sheet = 3)
```

For calculating alignment error rates, we only use the matrices containing retention times (RT). After conversion, all aligned datasets are in the same format, depicted below

```
knitr::kable(bfla_GCALIGNER[["aligned"]][["RT"]][1:10, 1:10],
    caption = "Alignment with GCALIGNER")
```

Table 1: Alignment with GCALIGNER

mean_RT	BFLA01	BFLA02	BFLA03	BFLA04	BFLA05	BFLA06	BFLA07	BFLA08	BFLA09
15.65627	15.664	15.632	15.665	15.664	15.669	15.657	15.668	15.655	15.630
16.32609	16.356	16.298	16.309	16.356	16.317	16.335	16.344	16.302	16.330
16.58973	16.597	16.583	16.587	16.597	16.588	16.591	16.594	16.584	16.589
16.93190	0.000	16.903	16.945	16.938	16.942	16.927	16.935	16.910	16.931
17.13760	0.000	17.112	0.000	0.000	0.000	17.151	17.143	17.137	17.145
17.16767	0.000	17.170	17.174	17.178	17.161	0.000	0.000	0.000	0.000
17.35655	17.356	17.376	17.354	17.356	17.355	17.354	17.355	17.352	17.354
17.49855	17.498	17.496	17.505	17.498	17.501	17.499	17.498	17.496	17.497
17.65482	17.656	17.655	17.655	17.656	17.655	17.654	17.656	17.653	17.653
18.13718	18.141	18.132	18.140	18.141	18.137	18.136	18.140	18.134	18.134

```
knitr::kable(bfla_GCalignR_optimised[["aligned"]][["RT"]][1:10,
    1:10], caption = "Alignment with GCalignR")
```

Table 2: Alignment with GCalignR

	mean_RT	BFLA01	BFLA02	BFLA03	BFLA04	BFLA05	BFLA06	BFLA07	BFLA08	BFLA09
2	15.65991	15.664	15.632	15.665	15.664	15.669	15.657	15.668	15.655	15.630
310	16.32973	16.356	16.298	16.309	16.356	16.317	16.335	16.344	16.302	16.330
4	16.59336	16.597	16.583	16.587	16.597	16.588	16.591	16.594	16.584	16.589
115	16.93590	0.000	16.903	16.945	16.938	16.942	16.927	16.935	16.910	16.931
118	17.14857	0.000	17.112	0.000	0.000	17.161	17.151	17.143	17.137	17.145
119	17.17600	0.000	17.170	17.174	17.178	0.000	0.000	0.000	0.000	0.000
5	17.36018	17.356	17.376	17.354	17.356	17.355	17.354	17.355	17.352	17.354
6	17.50218	17.498	17.496	17.505	17.498	17.501	17.499	17.498	17.496	17.497
7	17.65845	17.656	17.655	17.655	17.656	17.655	17.654	17.656	17.653	17.653
8	18.14082	18.141	18.132	18.140	18.141	18.137	18.136	18.140	18.134	18.134

Now we calculate alignment error rates for all datasets and for both tools.

```
df <- data.frame(software = rep(c("GCalignR", "GCALIGNER"), each = 3),
    dataset = rep(c("B. bimaculatus", "B. ephippiatus", "B. flavifrons"),
        2), error = c(error_rate(bbim_GCalignR_optimised, linshift = F,
```

```
        Reference = "data/bbim_ms.txt"), error_rate(beph_GCalignR_optimised,
        linshift = F, Reference = "data/beph_ms.txt"), error_rate(bfla_GCalignR_optimised,
        linshift = F, Reference = "data/bfla_ms.txt"), error_rate(bbim_GCALIGNER,
        linshift = F, Reference = "data/bbim_ms.txt"), error_rate(beph_GCALIGNER,
        linshift = F, Reference = "data/beph_ms.txt"), error_rate(bfla_GCALIGNER,
        linshift = F, Reference = "data/bfla_ms.txt")))
df$software <- factor(df$software, levels = c("GCalignR", "GCALIGNER"))
```

The alignment error rates stored in the data frame `df` are now visualised using a barplot

```
plot <- ggplot(df, aes(x = dataset, y = error, fill = software)) +
    geom_bar(stat = "identity", color = "black", position = position_dodge()) +
    labs(y = "Alignment error rate", x = "Bumblebee species") +
    theme_classic(base_size = 14) + theme(axis.text.x = element_text(face = "italic"),
    axis.title.x = element_text(margin = margin(t = 10, r = 20,
        b = 1, l = 0))) + scale_fill_manual(values = c("red",
    "darkblue")) + scale_y_continuous(breaks = seq(0, 0.05, 0.01),
    limits = c(0, 0.05), expand = c(0, 0))

print(plot)
```

Effects of parameter values on alignment results

To investigate the effects of different combinations of `max_diff_peak2mean` and `min_diff_peak2peak` on alignment error rates, we again used the three bumblebee datasets. We varied the parameter `max_diff_peak2mean` from 0.01 to 0.1 in steps of 0.01 and `min_diff_peak2peak` from 0.01 to 0.4 in steps 0.01. This results in 400 parameter combinations for which alignments are conducted using the code below.

```
# B. flavifrons
results_bfla <- optimal_params(data = "data/bfla.txt", rt_col_name = "RT",
    max_diff_peak2mean = seq(from = 0.01, to = 0.1, by = 0.01),
    min_diff_peak2peak = seq(from = 0.01, to = 0.4, by = 0.01))
save(results_bfla, file = "data/results_bfla.RData")

# B. bimaculatus
results_bbim <- optimal_params(data = "data/bbim.txt", rt_col_name = "RT",
    max_diff_peak2mean = seq(from = 0.01, to = 0.1, by = 0.01),
    min_diff_peak2peak = seq(from = 0.01, to = 0.4, by = 0.01))
save(results_bbim, file = "data/results_bbim.RData")

# B. ephippiatus
results_beph <- optimal_params(data = "data/beph.txt", rt_col_name = "RT",
    max_diff_peak2mean = seq(from = 0.01, to = 0.1, by = 0.01),
    min_diff_peak2peak = seq(from = 0.01, to = 0.4, by = 0.01))
save(results_beph, file = "data/results_beph.RData")
```

For each of the aligned datasets, alignment error rates are calculated using the function `error_rate` based on peaks that can be matched to identified substances according to supporting information Table S4 from Dellicour and Lecocq (2013). Errors are defined as shown in Eq 5 in the manuscript.

```
# Load data
load("data/results_bbim.RData")
load("data/results_beph.RData")
load("data/results_bfla.RData")
```

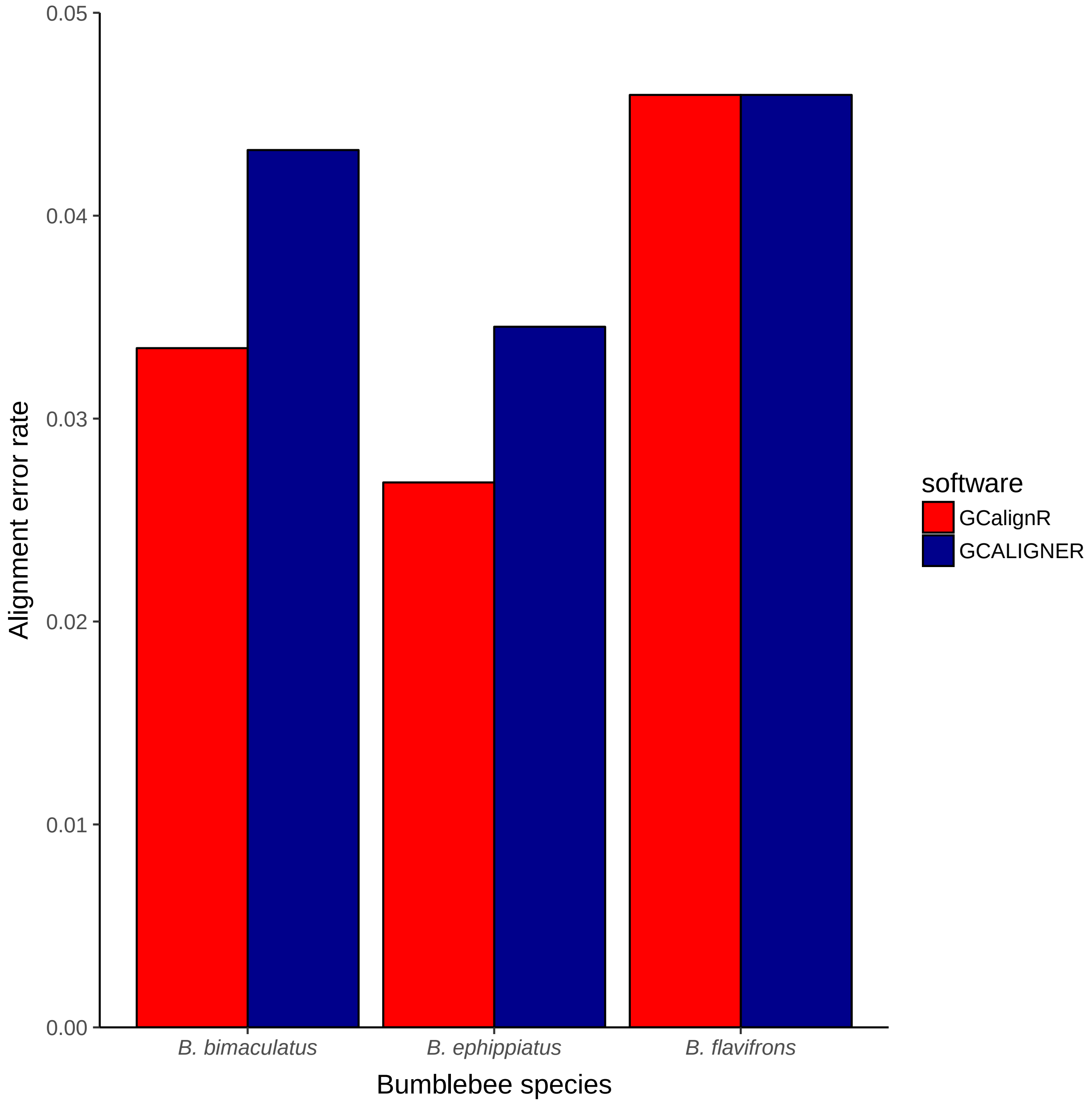

Figure 7: Alignment error rates for three bumblebee datasets using GCalignR and GCALIGNER.

```r
errors_bbim <- data.frame(p2p = results_bbim[[2]][["p2p"]], p2m = results_bbim[[2]][["p2m"]])

errors_bbim[["error"]] <- unlist(lapply(X = results_bbim[[1]],
    error_rate, "data/bbim_ms.txt"))

errors_beph <- data.frame(p2p = results_beph[[2]][["p2p"]], p2m = results_beph[[2]][["p2m"]])

errors_beph[["error"]] <- unlist(lapply(X = results_beph[[1]],
    error_rate, "data/beph_ms.txt"))

errors_bfla <- data.frame(p2p = results_bfla[[2]][["p2p"]], p2m = results_bfla[[2]][["p2m"]])

errors_bfla[["error"]] <- unlist(lapply(X = results_bfla[[1]],
    error_rate, "data/bfla_ms.txt"))

save(errors_bbim, file = "data/errors_bbim.RData")
save(errors_beph, file = "data/errors_beph.RData")
save(errors_bfla, file = "data/errors_bfla.RData")
```

The resulting alignment error rates can be visualised using three-dimensional plots

```r
with(errors_bbim, scatter3D(x = p2p, y = p2m, z = error, pch = 19,
    size = 2, theta = 30, phi = 0, ticktype = "detailed", main = "",
    xlab = "min_diff_peak2peak", ylab = "max_diff_peak2mean",
    zlab = "Error rate", bty = "g", colkey = FALSE, cex = 1,
    cex.lab = 1, cex.axis = 1, cex.main = 1.5, zlim = c(0, 0.2)))
```

```r
with(errors_beph, scatter3D(x = p2p, y = p2m, z = error, pch = 19,
    size = 2, theta = 30, phi = 0, ticktype = "detailed", main = "",
    xlab = "min_diff_peak2peak", ylab = "max_diff_peak2mean",
    zlab = "Error rate", bty = "g", colkey = FALSE, cex = 1,
    cex.lab = 1, cex.axis = 1, cex.main = 1.5, zlim = c(0, 0.2)))
```

```r
with(errors_bfla, scatter3D(x = p2p, y = p2m, z = error, pch = 19,
    size = 2, theta = 30, phi = 0, ticktype = "detailed", main = "",
    xlab = "min_diff_peak2peak", ylab = "max_diff_peak2mean",
    zlab = "Error rate", bty = "g", colkey = FALSE, cex = 1,
    cex.lab = 1, cex.axis = 1, cex.main = 1.5, zlim = c(0, 0.2)))
```

Comparison with parametric time warping

In order to compare alignments conducted with `GCalignR` to alignments using `ptw`, we use a dataset on European earwig (*Forficula auricularia*) individuals (Wong et al. 2014) that were downloaded from Dryad. This dataset is available as a text file 'earwig.txt' in the input format of `GCalignR`. In addition to retention time and peak intensity values this dataset contains the variable 'CHC' giving the substance identity of each peak (Wong et al. 2014), thereby allowing to calculate alignment error rates.

First, we begin to align the dataset with default parameter values in `GCalginR` using the following code.

```r
earwig_GCalignR_default <- align_chromatograms(data = "data/earwig.txt",
    rt_col_name = "RT")
save(earwig_GCalignR_default, file = "data/earwig_GCalignR_default.RData")
```

The alignment results saved to the file 'earwig_GCalignR_default.RData' are loaded to the workspace with

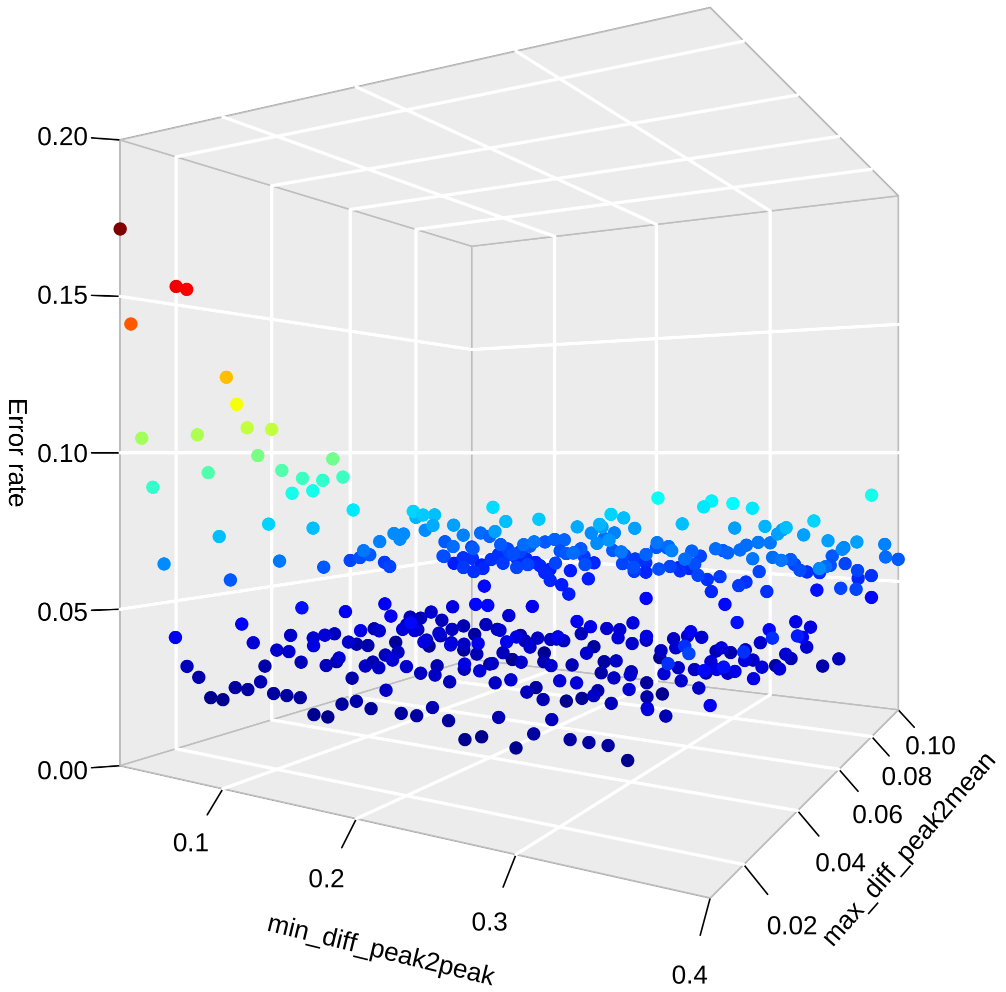

Figure 8: Effects of parameter combinations on alignment error rates based on the B. bimaculatus dataset.

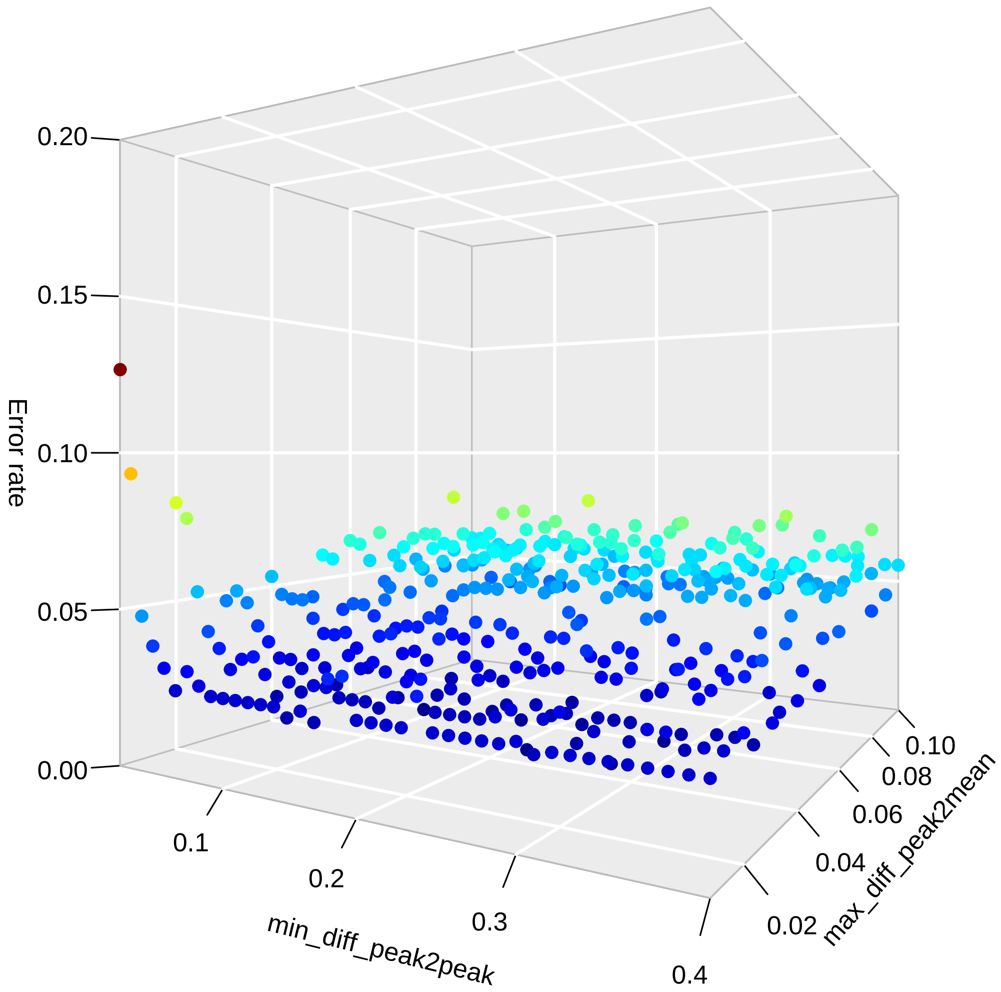

Figure 9: Effects of parameter combinations on alignment error rates based on the B. ephippiatus dataset.

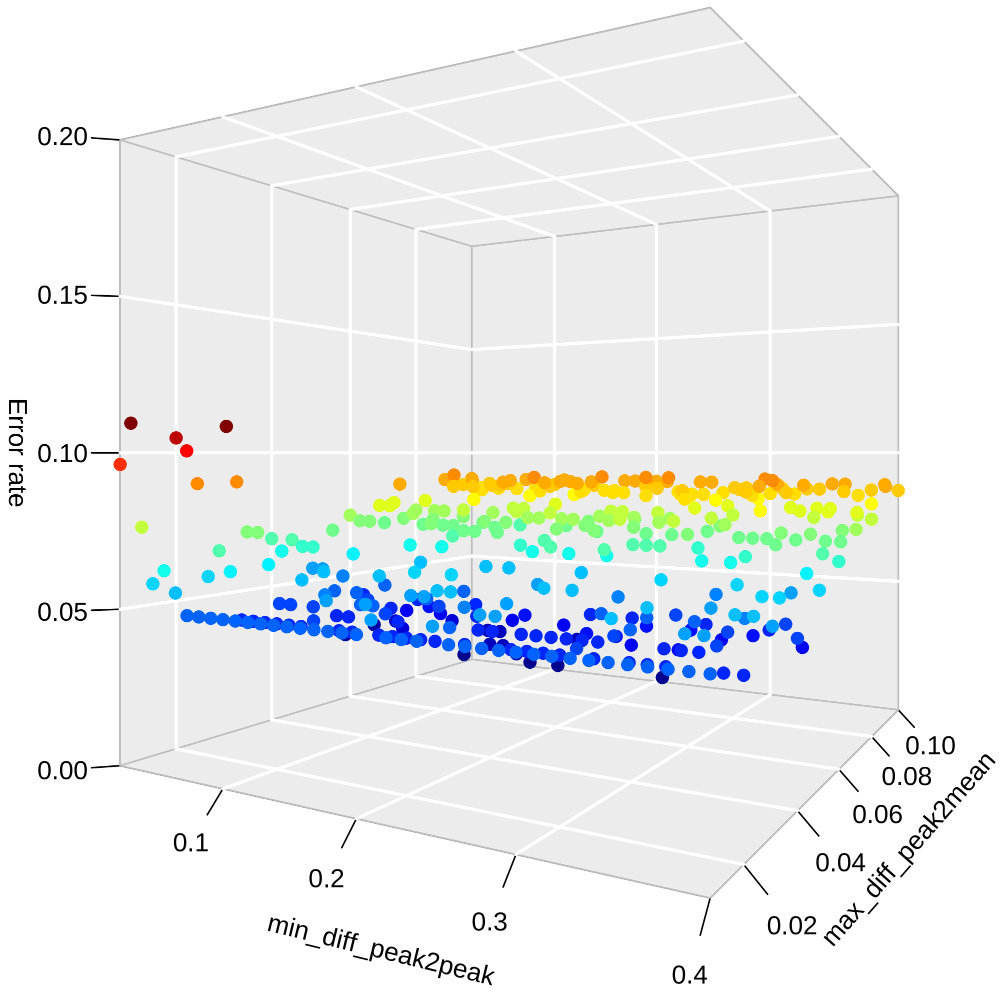

Figure 10: Effects of parameter combinations on alignment error rates based on the B. flavifrons dataset.

```
load("data/earwig_GCalignR_default.RData")
```

As shown already above for the alignment of the bumblebee datasets, we inspect the alignment results with diagnostic plots.

```
plot(earwig_GCalignR_default, which_plot = "shifts", main = "Distribution of linear shift in the alignm
```

The distribution of applied linear shifts indicates that a small proportion of samples required a linear transformation saturating the allowed window size of -0.02 to 0.02. Therefore, we will increase the parameter value of `max_linear_shift` to 0.05 in the fine-tune alignment to ensure that all linear shifts can be corrected. Additionally, we inspect alignments with the heatmap function `gc_heatmap`.

```
gc_heatmap(earwig_GCalignR_default, main = "Alignment with default parameters",
    label = "x", label_size = 12, show_legend = F, samples_subset = 1:200)
```

The heatmap for a subset of 200 samples, shows distinct patterns for multiple adjacent substances that require increasing the value of `min_diff_peak2peak` in order to merging the putatively homologous substances. For instance substances annotated by '29.86', '29.99' and '30.36' appear to represent all the same homologous substance with highly variable retention times for a subset of the dataset. Hence, we fine-tune the alignment by setting `min_diff_peak2peak = 0.75`

```
earwig_GCalignR_optimised <- align_chromatograms(data = "data/earwig.txt",
    rt_col_name = "RT", max_linear_shift = 0.05, min_diff_peak2peak = 0.75)
save(earwig_GCalignR_optimised, file = "data/earwig_GCalignR_optimised.RData")
```

```
load("data/earwig_GCalignR_optimised.RData")
```

By inspecting the distribution of applied linear shifts in the fine-tuned alignment, we ensure that now all systematic shifts in retention times have been accounted for.

```
plot(earwig_GCalignR_optimised, which_plot = "shifts", main = "Distribution of linear shift in the fine
```

Also we inspect the heatmap again

```
gc_heatmap(earwig_GCalignR_optimised, main = "Alignment with fine/tuned parameters",
    label = "x", label_size = 12, show_legend = F, samples_subset = 1:200)
```

Both plots suggest that the fine-tuning of the alignment parameter resulted in an adequate alignment of the dataset. In addition to the retention time values, two other variables ('Response' and 'CHC') were aligned as well denoting peak intensity and peak identity respectively. This allows to estimate the error rate of the alignment conducted with `GCalignR`. From the `print` function we know that the expected number of 20 substances are aligned in the dataset

```
print(earwig_GCalignR_optimised)
#> Summary of Peak Alignment running align_chromatograms
#> Input: data/earwig.txt
#> Start:  2017-09-11 15:07:37  Finished:  2017-09-11 15:12:11
#>
#> Call:
#>   GCalignR::align_chromatograms(data=data/earwig.txt, rt_col_name=RT,
#>   max_linear_shift=0.05, min_diff_peak2peak=0.75, sep=\t, rt_cutoff_low=NULL,
#>   rt_cutoff_high=NULL, reference=NULL, max_diff_peak2mean=0.02,
#>   delete_single_peak=FALSE)
#>
#> Summary of scored substances:
#>    total retained
#>       20       20
```

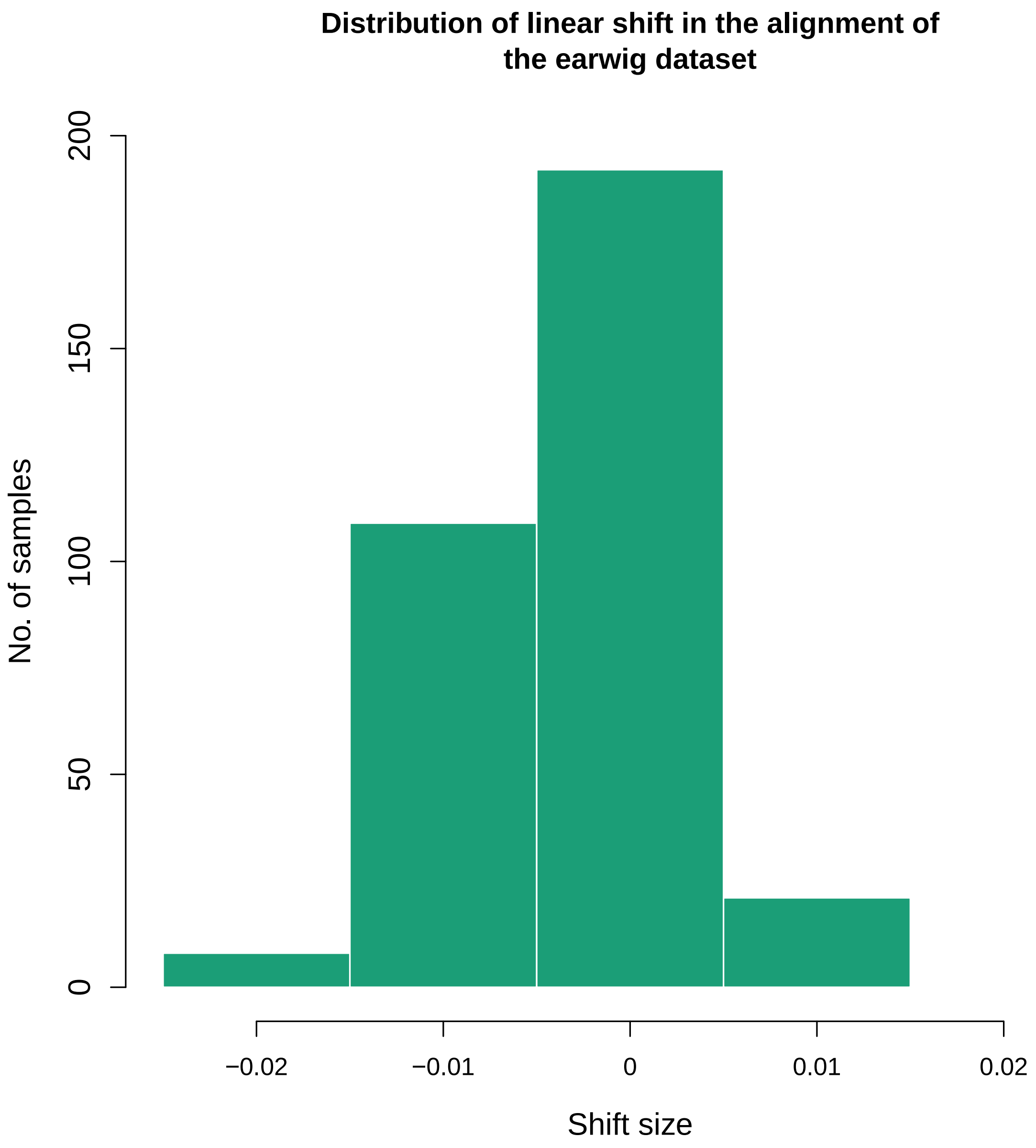

Figure 11: Left skewed distribution of applied linear shifts during the first step of the alignment procedure for an earwig dataset suggest to increase the window size used for the alignment.

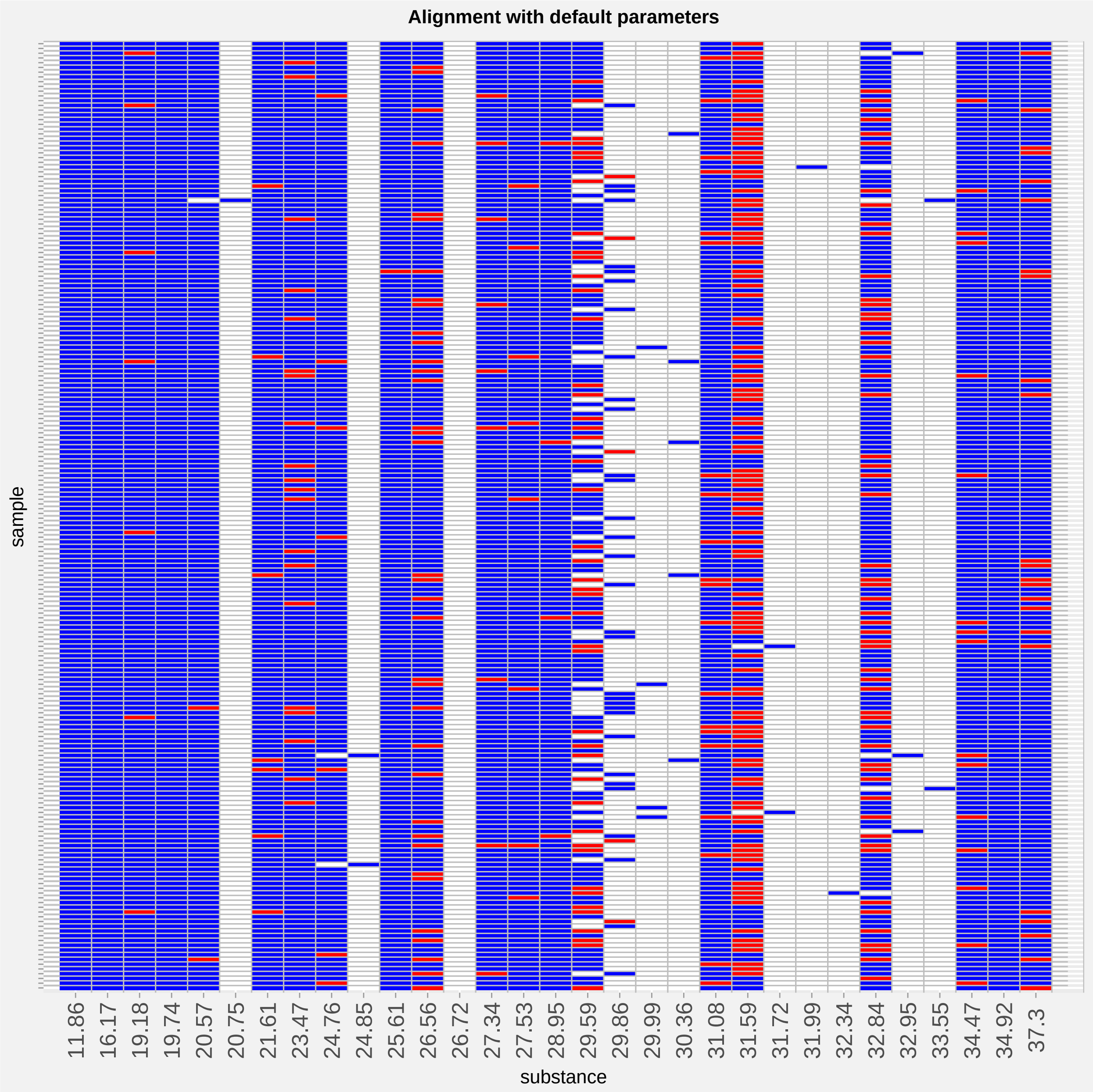

Figure 12: Heatmap showing the distribution of substances across the first 200 samples of the dataset.

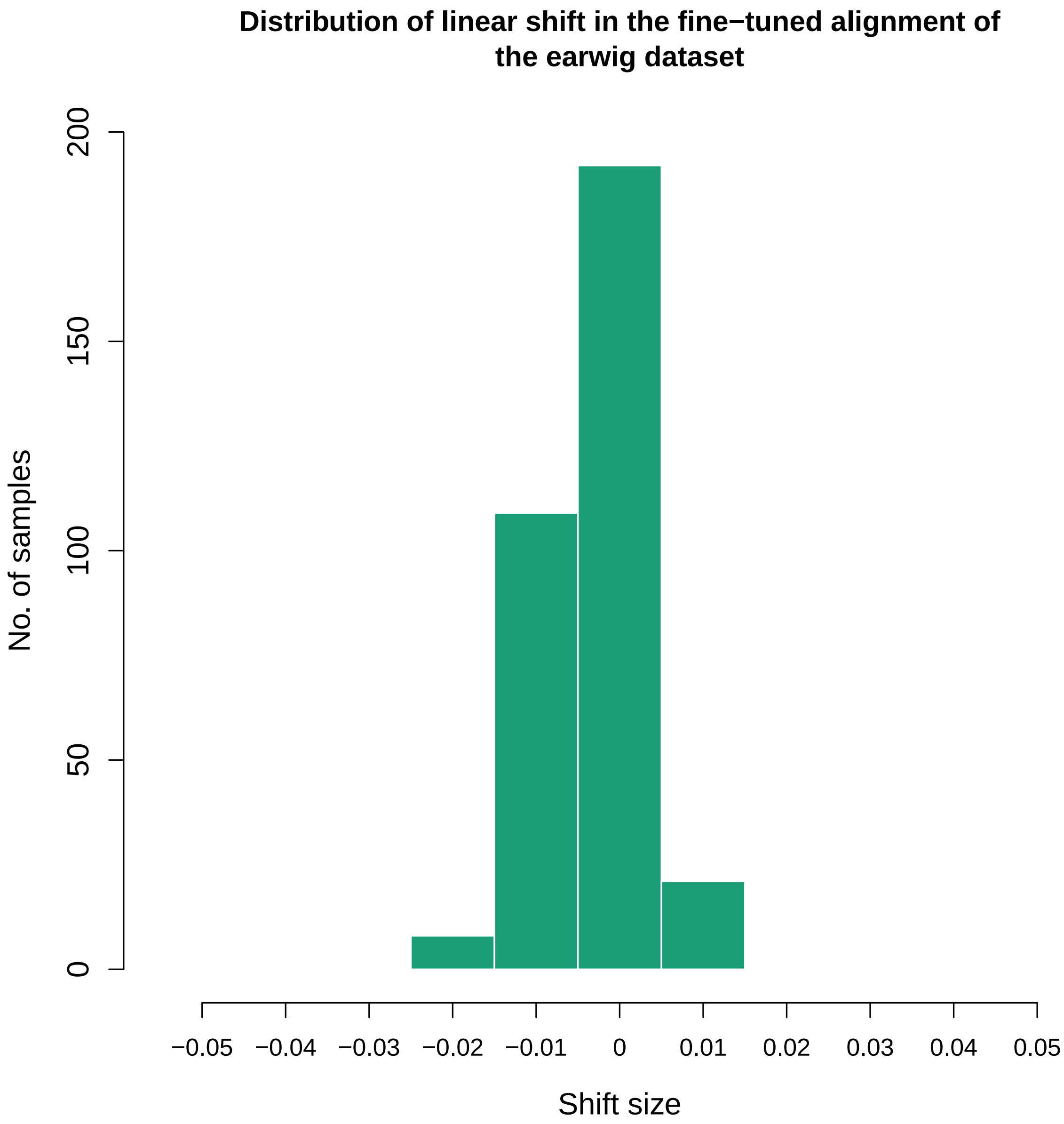

Figure 13: Distribution of linear shifts applied during the first step of the alignment procedure implemented in GCalignR. All applied shifts are centered around zero and are and do not extend to the entire range allowed by the window size.

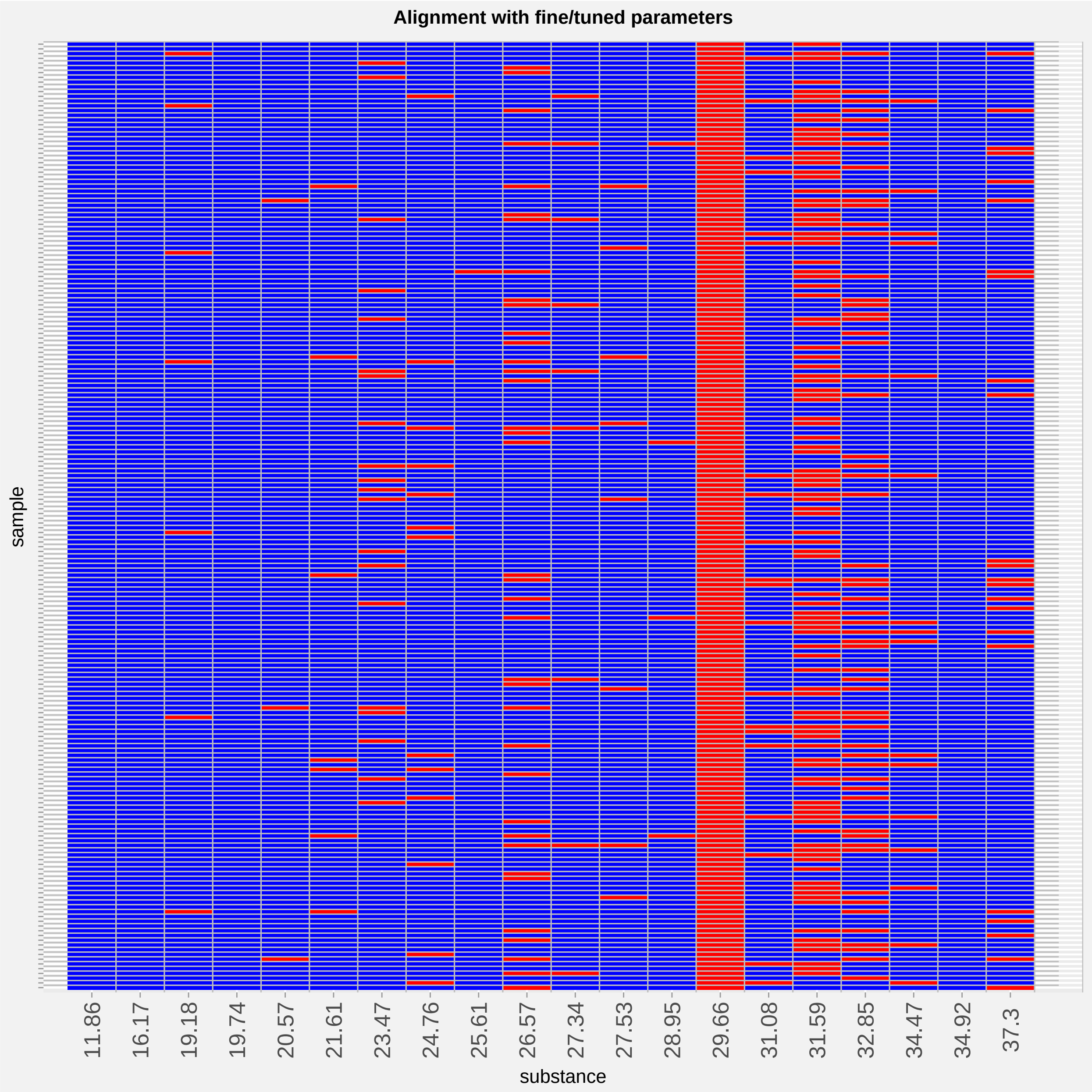

Figure 14: Heatmap showing the distribution of substances in the earwig dataset after fine-tuning alignment parameters.

```
#>
#> In total 20 substances were identified among all samples. 20 substances are
#>   retained after all filtering steps.
#>
#> Sample overview:
#>   The following 330 samples were aligned to the reference 'Ad_001':
#>   Ad_001, Ad_002, Ad_003, Ad_004, Ad_005, Ad_006, Ad_007, Ad_008, Ad_009, Ad_010,
#>   Ad_011, Ad_012, Ad_013, Ad_014, Ad_015, Ad_016, Ad_017, Ad_018, Ad_019, Ad_020,
#>   Ad_021, Ad_022, Ad_023, Ad_024, Ad_025, Ad_026, Ad_027, Ad_028, Ad_029, Ad_030,
#>   Ad_031, Ad_032, Ad_033, Ad_034, Ad_035, Ad_036, Ad_037, Ad_038, Ad_039, Ad_040,
#>   Ad_041, Ad_042, Ad_043, Ad_044, Ad_045, Ad_046, Ad_047, Ad_048, Ad_049, Ad_050,
#>   Ad_051, Ad_052, Ad_053, Ad_054, Ad_055, Ad_056, Ad_057, Ad_058, Ad_059, Ad_060,
#>   Ad_061, Ad_062, Ad_063, Ad_064, Ad_065, Ad_066, Ad_067, Ad_068, Ad_069, Ad_070,
#>   Ad_071, Ad_072, Ad_073, Ad_074, Ad_075, Ad_076, Ad_077, Ad_078, Ad_079, Ad_080,
#>   Ad_081, Ad_082, Ad_083, Ad_084, Ad_085, Ad_086, Ad_087, Ad_088, Ad_089, Ad_090,
#>   Ad_091, Ad_092, Ad_093, Ad_094, Ad_095, Ad_096, Ad_097, Ad_098, Ad_099, Ad_100,
#>   Ad_101, Ad_102, Ad_103, Ad_104, Ad_105, Ad_106, Ad_107, Ad_108, Ad_109, Ad_110,
#>   Ad_111, Ad_112, Ad_113, Ad_114, Ad_115, Ad_116, Ad_117, Ad_118, Ad_119, Ad_120,
#>   Ad_121, Ad_122, Ad_123, Ad_124, Ad_125, Ad_126, Ad_127, Ad_128, Ad_129, Ad_130,
#>   Ad_131, Ad_132, Ad_133, Ad_134, Ad_135, Ad_136, Ad_137, Ad_138, Ad_139, Ad_140,
#>   Ad_141, Ad_142, Ad_143, Ad_144, Ad_145, Ad_146, Ad_147, Ad_148, Ad_149, Ad_150,
#>   Ad_151, Ad_152, Ad_153, Ad_154, Ad_155, Ad_156, Ad_157, Ad_158, Ad_159, Ad_160,
#>   Ad_161, Ad_162, Ad_163, Ad_164, Ad_165, Ad_166, Ad_167, Ad_168, Ad_169, Ad_170,
#>   Ad_171, Ad_172, Ad_173, Ad_174, Ad_175, Ad_176, Ad_177, Ad_178, Ad_179, Ad_180,
#>   Ad_181, Ad_182, Ad_183, Ad_184, Ad_185, Ad_186, Ad_187, Ad_188, Ad_189, Ad_190,
#>   Ad_191, Ad_192, Ad_194, Ad_195, Ad_196, Ad_197, Ad_198, Ad_199, Ad_200, Ad_201,
#>   Ad_202, Ad_203, Ad_204, Ad_205, Ad_206, Ad_207, Ad_208, Ad_209, Ad_210, Ad_211,
#>   Ad_212, Ad_213, Ad_214, Ad_215, Ad_216, Ad_217, Ad_218, Ad_219, Ad_220, Ad_221,
#>   Ad_222, Ad_223, Ad_224, Ad_225, Ad_226, Ad_227, Ad_228, Ad_229, Ad_230, Ad_231,
#>   Ad_232, Ad_233, Ad_234, Ad_235, Ad_236, Ad_237, Ad_238, Ad_239, Ad_240, Ad_241,
#>   Ad_242, Ad_243, Ad_244, Ad_245, Ad_246, Ad_247, Ad_248, Ad_249, Ad_250, Ad_251,
#>   Ad_252, Ad_253, Ad_254, Ad_255, Ad_256, Ad_257, Ad_258, Ad_259, Ad_260, Ad_261,
#>   Ad_262, Ad_263, Ad_264, Ad_265, Ad_266, Ad_267, Ad_268, Ad_269, Ad_270, Ad_271,
#>   Ad_272, Ad_273, Ad_274, Ad_275, Ad_276, Ad_277, Ad_278, Ad_279, Ad_280, Ad_281,
#>   Ad_282, Ad_284, Ad_285, Ad_286, Ad_287, Ad_288, Ad_289, Ad_290, Ad_291, Ad_292,
#>   Ad_293, Ad_294, Ad_295, Ad_296, Ad_297, Ad_298, Ad_299, Ad_300, Ad_301, Ad_302,
#>   Ad_304, Ad_306, Ad_307, Ad_308, Ad_309, Ad_310, Ad_311, Ad_313, Ad_314, Ad_315,
#>   Ad_316, Ad_317, Ad_318, Ad_319, Ad_320, Ad_321, Ad_322, Ad_323, Ad_324, Ad_326,
#>   Ad_327, Ad_328, Ad_329, Ad_330, Ad_331, Ad_332, Ad_333, Ad_334, Ad_335, Ad_336
#>
#> For further details type:
#>   'gc_heatmap(earwig_GCalignR_optimised)' to retrieve heatmaps
#>   'plot(earwig_GCalignR_optimised)' to retrieve further diagnostic plots
```

Therefore, we simply evaluate that each aligned substance in the dataset is only comprised of the same peak identity using the aligned matrix for the variable 'CHC'. A subset of the matrix is shown below. Each value of the matrix correspond to a hydrocarbon peak identified in Wong et al. 2014, except for '99' that encodes the internal standard used for this GC-MS dataset.

```
knitr::kable(earwig_GCalignR_optimised$aligned$CHC[1:10, 1:10],
    caption = "Alignment with GCalignR")
```

Table 3: Alignment with GCalignR

	mean_RT	Ad_001	Ad_002	Ad_003	Ad_004	Ad_005	Ad_006	Ad_007	Ad_008	Ad_009
1	11.86428	99	99	99	99	99	99	99	99	99
2	16.17371	6	6	6	6	6	6	6	6	6
3	19.18487	10	10	10	10	10	10	10	10	10
41	19.74183	11	11	11	11	11	11	11	11	11
5	20.57094	12	12	12	12	12	12	12	12	12
6	21.60952	13	13	13	13	13	13	13	13	13
7	23.47025	14	14	14	14	14	14	14	14	14
8	24.76218	15	15	15	15	15	15	15	15	15
9	25.60964	16	16	16	16	16	16	16	16	16
10	26.56522	17	17	17	17	17	17	17	17	17

In order to test that each row only contains the same value, we calculate rowise standard deviations that will be zero when all values are the same.

```
apply(earwig_GCalignR_optimised$aligned$CHC[-1], 1, sd)
#>  1  2  3 41  5  6  7  8  9 10 11 12 13 14 15 16 17 18 19 20
#>  0  0  0  0  0  0  0  0  0  0  0  0  0  0  0  0  0  0  0  0
```

The output above shows that `GCalignR` aligned the dataset without any error following fine-tuning of alignment parameters.

Testing the performance of ptw

Next, we use the same earwig dataset to align retention times using the package ptw. `ptw` supports to use a peak list comprising two variables containing retention time values and intensities of peaks named as *rt* and *I* respectively. Therefore we use the function `convert2ptw` to convert the text file 'earwig.txt' that contains the chemical dataset accordingly.

```
earwig_ptw_input <- convert2ptw(data = "data/earwig.txt", rt_col_name = "RT",
    conc_col_name = "Response")
```

The format of the input file for `ptw` is shown below using R summary functions.

```
class(earwig_ptw_input)  # a list of samples
#> [1] "list"
str(earwig_ptw_input$Ad_001)  # each list contains a matrix
#>  num [1:20, 1:2] 11.9 16.2 19.2 19.7 20.6 ...
#>  - attr(*, "dimnames")=List of 2
#>   ..$ : chr [1:20] "1" "2" "3" "4" ...
#>   ..$ : chr [1:2] "rt" "I"
```

`ptw` offers a function called 'stptw' that implements parametric time warping of a peak list and requires a user-defined reference sample (Wehrens et al. 2015). Therefore, we specify the first sample in earwig dataset 'Ad_001' as a reference for the retention time alignment and extract the corresponding peak data from the dataset.

```
ref <- "Ad_001"
index <- which(names(earwig_ptw_input) == ref)  # position in the dataset
refst <- earwig_ptw_input[index]  # extract the reference
sampst <- earwig_ptw_input[-index]  # remove the reference from the list of samples
```

Before conducting the alignment with `ptw` we optimise the WCC criterion (Bloemberg et al. 2013, Wehrens et al. 2015), expressed by the parameter `trwdth`. See `?stptw` for details. Based on the knowledge that all peaks are shared between reference and sample. We can pick the value of `trwdth` that yields to best alignment performance defined as the smallest summed deviation in retention times between the reference and the first sample of the dataset.

```
## preallocate a data frame to store results
opt.crit <- data.frame(wcc = 0, sum_dev = 0)
## set up a vector of values to test
wcc <- c(seq(0.1, 0.9, 0.1), 1:100)
## loop over all values
for (i in 1:length(wcc)) {
    ## apply the warping
    ptw_out <- stptw(refst, sampst[1], trwdth = wcc[i])
    ## extract results
    opt.crit[i, ] <- c(wcc[i], sum(abs(ptw_out$warped.sample[[1]][,
        1] - ptw_out$reference[[1]][, 1])))
}
```

The optimal value of the parameter `trwdth` is given in the first row of the following table

```
head(opt.crit[order(opt.crit$sum_dev, decreasing = F), ])
#>    wcc  sum_dev
#> 11 2.0 1.279437
#> 12 3.0 1.334910
#> 7  0.7 1.445403
#> 5  0.5 1.446737
#> 8  0.8 1.446739
#> 9  0.9 1.451569
```

Using a value of `trwdth = 2` we perform the alignment in `ptw` using the following code

```
ptw_out <- lapply(sampst, function(x) stptw(refst, list(x), trwdth = 2))
```

The out contains the input retention time values of all peaks as well as aligned retention times for each of the samples. These allow to calculate deviations in retention times between homologous peaks with respect to the reference 'Ad_001'.

```
## Estimate deviations
aligned <- do.call("rbind", lapply(ptw_out, function(fx) {
    temp <- abs(as.vector(fx$warped.sample[[1]][, 1]) - as.vector(fx$reference[[1]][,
        1]))
}))

## Obtain deviation from the raw data
input <- do.call("rbind", lapply(ptw_out, function(fx) {
    temp <- abs(as.vector(fx$sample[[1]][, 1]) - as.vector(fx$reference[[1]][,
        1]))
}))

## get peak names for cross-reference to Wong et al. 2014
peaks <- c("IS", as.character(read_peak_list(data = "data/earwig.txt",
    rt_col_name = "RT")[[1]][2:20, 1]))

## convert to data frames
df1 <- as.data.frame(aligned)
names(df1) <- peaks
```

```r
df1 <- suppressMessages(reshape2::melt(df1))
df2 <- as.data.frame(input)
names(df2) <- peaks
df2 <- suppressMessages(reshape2::melt(df2))


## calculate mean and standard deviations
df <- data.frame(var = rep(df1$variable, 2), val = c(0 - df2$value,
    df1$value - df2$value), software = rep(c("GCalignR", "ptw"),
    each = nrow(df1)))
```

The data frame 'df' contains the deviation scores for each of the 20 peaks prior to and after aligning the dataset with `ptw`. The deviations values are plotted using the following code

```r
plot <- ggplot(df, aes(x = var, y = val, fill = software)) +
    geom_boxplot(outlier.colour = "white") + labs(y = "Change in retention time deviation [minutes]",
    x = "Substances") + theme_classic(base_size = 12) + scale_fill_manual(values = c("red",
    "blue")) + scale_y_continuous(expand = c(0, 0), limits = c(-0.15,
    0.25), breaks = seq(-0.25, 0.25, 0.05)) + geom_hline(yintercept = 0,
    linetype = "dashed")

print(plot)
```

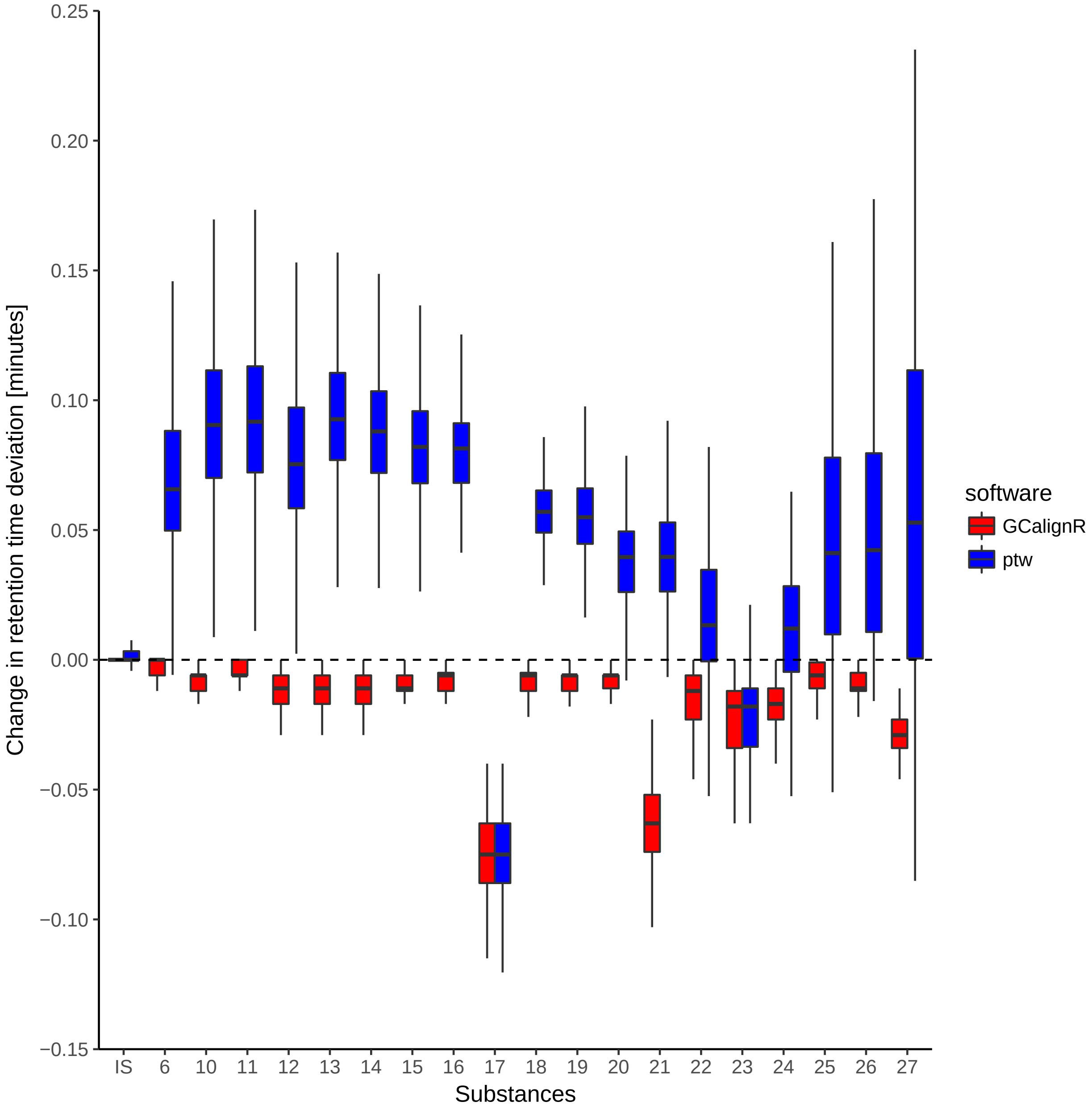

Figure 15: Performance of the alignment algorithm implemented in ptw.

References

Bloemberg, T. G., Gerretzen, J., Wouters, H. J., Gloerich, J., van Dael, M., Wessels, H. J., … & Wehrens, R. (2010). Improved parametric time warping for proteomics. Chemometrics and Intelligent Laboratory Systems, 104(1), 65-74.

Dellicour S, Lecocq T. GCALIGNER 1.0: an alignment program to compute a multiple sample comparison data matrix from large eco-chemical dataset obtained by GC. Journal of separation science. 2013;36(19):3206-3209. doi:10.1002/jssc.201300388

Oksanen J, Blanchet FG, Friendly M, Kindt R, Legendre P, McGlinn D, et al.. vegan: Community Ecology Package; 2016. Available from: https://CRAN.R-project.org/package=vegan.

Wehrens, Ron, Tom G. Bloemberg, and Paul HC Eilers. "Fast parametric time warping of peak lists." Bioinformatics 31.18 (2015): 3063-3065.

Wickham H. ggplot2: Elegant Graphics for Data Analysis. Springer-Verlag New York; 2009. Available from: http://ggplot2.org.

Wong JWY, Meunier J, Lucas C, Kölliker M. Paternal signature in kin recognition cues of a social insect: concealed in juveniles, revealed in adults. Proceedings of the Royal Society of London B: Biological Sciences. 2014;281(1793):20141236.
