# Supplementary material for: GCalignR: An R package for aligning gas-chromatography data for ecological and evolutionary studies
Source: PLoS One. 2018 Jun 7;13(6):e0198311. doi: 10.1371/journal.pone.0198311 (PMC5991698; doi:10.1371/journal.pone.0198311)
Supplement: S4 File — (HTML) [file pone.0198311.s004.html]

GCalignR Step by Step


# GCalignR Step by Step

#### *Meinolf Ottensmann, Martin A. Stoffel, Hazel J. Nichols and Joseph I. Hoffman*

## Introduction

We developed `GCalignR` primarily as an alignment tool for GC-FID data based on peak retention times, but other types of data that contain retention time information for peaks (e.g. GC-MS) are supported as well. `GCalignR` implements a fast and objective method to cluster putatively homologous substances prior to multivariate statistical analyses. Using sophisticated visualisations, the resulting alignments can then be fine tuned. The input format of `GCalignR` is a peak list, which is comprised of retention times and arbitrary variables (e.g. peak height, peak area) that characterise each peak in a dataset.

The implemented algorithm is purely based on retention time data, which is why the quality of the alignment is highly dependent on the quality of the raw data and the parameters used for the initial peak detection. In other words: The clearer the peaks are which were extracted from the chromatograms, the better the alignment will be. `GCalignR` has been created for situations where the main interest of the research is in exploring broader patterns rather then the specific function of a certain chemical, which is unlikely to be determined correctly in all cases when just retention times are used. Also, we recommend to double-check the resulting alignment with mass-spectrometry data, where available. Furthermore, replicates that were analysed using both GC-MS and GC-FID with identical gas chromatography settings can be aligned together and may be used to identify peaks and validate alignment results.

This vignette gives an quick introduction into using `GCalignR`, whereas a more detailed description of the background is found in our manuscript(Ottensmann et al. 2017) and in the second vignette of the package *“GCalignR: How does the Algorithm work?”*.

## GCalignR workflow in a larger context

In the flow diagram below, we visualized the functionality of GCalignR within a complete workflow of analysing chemical data. After (1) analysis of the chemical samples with GC-FID, an often proprietary software is used to extract a list of peaks (retention times, peak area, also often peak height and other variables). Steps (3)-(7) are the alignment steps within `GCalignR`, detailed below. After alignment and normalisation, the output can be used as input for multivariate statistics in other packages such as vegan (8).

Extended Workflow using GCalignR in the analysis of chemical similarity patterns.

## Installation

The development version can be downloaded from GitHub with the following code:

```
install.packages("devtools") 
devtools::install_github("mottensmann/GCalignR", build_vignettes = TRUE)
```

```
library("GCalignR")
```

The package documentation can be accessed with:

```
?GCalignR # documentation
```

## The functions below form the core of `GCalignR`:

- `align_chromatograms`: The entire alignment procedure is implemented within this function based on three parameter values described below. Additional parameters allow optional processing steps.
- `plot.GCalign`: Diagnostic plots summarise the aligned dataset.
- `print.GCalign`: Summarises the alignment process and lists all arguments to the function call.
- `gc_heatmap`: Visualises alignment results and enables to quickly inspect the distribution of substances (i.e aligned peaks) within the dataset. Furthermore, the deviation from mean retention times of a given substance can be used to detect potential issues with the alignment.
- `check_input`: Checks the format of the input data for conformity with the requirements and highlights violations.
- `norm_peaks`: Normalises the abundance measure of peaks by calculating relative intensities within samples.
- `as.data.frame.GCalign`: Outputs aligned datasets within a list of data frames for each variable in the input data.

## The alignment algorithm

We developed an alignment procedure that involves three sequential steps to align and finally match peaks belonging to putatively homologous substances across samples. The first step is to align each sample to a reference sample while maximising overall similarity through linear shifts of retention times. This procedure is often described in the literature as ‘full alignment’. In the second step, individual peaks are sorted into rows based on close similarity of their retention times, a procedure that is often referred to as ‘partial alignment’. Finally, there is still a chance that homologous peaks can be sorted into different, but adjacent, rows in different samples, depending on the variability of their retention times Consequently, a third step merges rows representing putatively homologous substances.

The alignment algorithm implemented in the `align_chromatograms` function contains the following steps: (Here we refer to a peak list as all extracted peaks from a given sample chromatogram)

1. The first step in the alignment procedure consists of an algorithm that corrects systematic linear shifts between peaks of a query sample and a fixed reference to account for systematic shifts in retention times among samples. By default the sample that is most similar on average to all other samples is automatically selected as reference. With respect to the user-defined parameter `max_linear_shift` linear shifts shifts are applied to all retention times of a sample to maximise similarity to the reference.
2. The second step in the alignment procedure aligns individual peaks across samples by comparing the peak retention times of each sample consecutively with the mean of all previous samples The parameter `max_diff_peak2mean` specifies the allowed deviation between the retention time of a peak and the mean of previous retention times within the same row. If the deviation is larger than allowed, matrix operations are conducted to sort the peaks accordingly.

3.The third step in the alignment procedure accounts for the fact that a number of homologous peaks will be sorted into multiple rows that can be subsequently merged The maximum mean difference between two retention time rows can be specified with the `min_diff_peak2peak` argument.

### Optional steps:

4. Delete peaks that occur in just one sample by setting the `delete_single_peak` argument to `TRUE`
5. Delete all peaks that occur in negative control samples by specifying their names as argument to `blanks`

## Input data

The statistical analysis of GC-FID or GC-MS data is usually based on the detection of peaks (i.e. substances) within chromatograms instead of using the whole profile. Peak can be integrated using proprietary software or free programs. The peak data derived from a chromatogram usually contains the retention time of a given peak plus additional information such as the area under the peak or its height which are used in the subsequent analysis. `GCalignR` uses only the retention times (and not the mass-spectra, which may not be available, e.g. when using gas-chromatography coupled to a flame ionization detector (FID)) to align the peaks across individuals for subsequent chemometric analysis and pattern detection. The simple assumption is that peaks with similar retention times represent the same substances. However, it is recommended to verify this assumption by comparing also the mass-spectra (if available) of the substances of interest. The input peak list used in `GCalignR` is a plain text file, whereby all elements should be separated by tabs (with sep = “/t”) or any other separator, which has to be specified with the `sep` argument (see `?read.table` for a list of separators). The decimal separator has to be the point.

- The first row contains the sample names, whereby every name must be unique. Names should not contain whitespaces and use the underscore as the only special character.
- The second row contains the names of the data columns. For example, if both the retention time of a peak as well as the area under the peak have been extracted you would write `RT area` into the second line. You can however add more variables such as the peak height. The variable names should not contain whitespaces and use the underscore as the only special character.
- Starting with the third row, the actual peak data is included, whereby single samples are concatenated horizontally. Each chromatogram needs to consist of the same number of columns, whereby at least one containing retention times is required. In order to use the functionality of the function `norm_peaks` an additional column containing a concentration measure (e.g. height or area of peaks) is required. The decimal separator has to be the point (not comma!).

Example of a input datafile. Only the head of the file is shown.

Naturally, not all chromatograms contain the same number of peaks.

Tail of the input file

### Alternative input from R

Alternatively to reading a text file, `GCalignR` also accepts lists of data frames. The name of each list element corresponds to the identity of a sample and the data frame contains the peak data for this sample (again, the minimum number of columns is one column for the retention time and one column for another variable such as the area under the peak or it’s height is required for using `norm_peaks`). All column names within the data frames have to be the same and named consistently. The attached dataset `peak_data` contains data from skin swabs of Antarctic Fur Seals *Arctocephalus gazella* (Stoffel et al. 2015) and can be used as template.

```
data("peak_data")
length(peak_data) # number of individuals, i.e. number of list elements
#> [1] 84
names(peak_data) # names of individuals, i.e. names of list elements 
#>  [1] "C3"  "C2"  "M2"  "M3"  "M4"  "M5"  "M6"  "M7"  "M8"  "M9"  "M10"
#> [12] "M12" "M14" "M15" "M16" "M17" "M18" "M19" "M20" "M21" "M23" "M24"
#> [23] "M25" "M26" "M27" "M28" "M29" "M30" "M31" "M33" "M35" "M36" "M37"
#> [34] "M38" "M39" "M40" "M41" "M43" "M44" "M45" "M46" "M47" "M48" "P2" 
#> [45] "P3"  "P4"  "P5"  "P6"  "P7"  "P8"  "P9"  "P10" "P12" "P14" "P15"
#> [56] "P16" "P17" "P18" "P19" "P20" "P21" "P23" "P24" "P25" "P26" "P27"
#> [67] "P28" "P29" "P30" "P31" "P33" "P35" "P36" "P37" "P38" "P39" "P40"
#> [78] "P41" "P43" "P44" "P45" "P46" "P47" "P48"
head(peak_data[[1]]) # column names and data, i.e. one data.frame of list element 
#>   time     area
#> 1 4.53  3331224
#> 2 4.55  1462381
#> 3 4.62  4834211
#> 4 4.68  7754401
#> 5 4.71  1267617
#> 6 4.79 10356487
```

## GCalignR workflow

### Check the input

To check the data formatting for the most common errors, use the `check_input` function. This will test for conformity with the the main requirements of the aligning algorithm and give a warning message if these aren’t met. When a text file is used as input, the decimal has to be a point (not a comma). However, there could potentially be a variety of different error sources so it is advisable to make sure by yourself that the data is in the correct format.

```
# if plot = T, a histogram of peaks is plotted
check_input(data = peak_data,plot = F)  
#> All checks passed!
```

The alignment procedure adjust peaks that represent putatively homologous substances that differ to varying degree in retention times among samples. In order to define sensible values for the the parameters `min_diff_peak2peak` it is advisable to investigate how far peaks are commonly separated within chromatograms. The function `peak_interspace` plots a histogram of all between peak retention time differences within samples and combines this estimates for the complete dataset. Here, the difference between close peaks is of interest and and the function allows to plot certain quantile ranges of the distribution by specifying the parameter `quantile_range`.

```
peak_interspace(data = peak_data, rt_col_name = "time",
                quantile_range = c(0, 0.8), quantiles = 0.05)
```

Figure 4: Histogram of Peak interspaces

```
#> $Summary
#>    Min. 1st Qu.  Median    Mean 3rd Qu.    Max. 
#>  0.0000  0.0600  0.1000  0.2542  0.2500 11.3700 
#> 
#> $Quantiles
#>   5% 
#> 0.03
```

The histogram shows the distribution of retention-time ‘spaces’ between peaks. Most peaks are around 0.05 minutes apart from each other. From the distribution we want to infer the potential error-margin around a peak,that GCalignR will correct. We can see from the histogram that very few peaks are closer together than 0.03 minutes. By looking at the original chromatograms it becomes clear that these peaks are usually substances with low concentration which show a ‘double peak’, i.e. two peaks appear for what we believe is just a single substance. Therefore, we decide to take a value of 0.03 for the `min_diff_peak2peak` parameter below. It is noteworthy that this does not set a strict threshold and substances with a smaller difference in mean retention times can still be formed during the alignment (because those peaks are known to exist within samples). Therefore, we suggest to carefully check the aligned data and revise the initial peak calling if required.

### Align chromatograms

The core function in `GCalignR` is `align_chromatograms`, which will align the peak lists with the algorithm described above. See `?align_chromatograms` for a detailed description of the arguments. The alignment process will take a few minutes to several hours on a standard computer depending on mainly three factors; (1) the number of samples and (2) the number of peaks per sample and (3) the number of substances that are classified during the alignment procedure. Progress bars and run time estimates (where possible) indicate the progress of the alignment run.

```
peak_data <- peak_data[1:4] # subset for speed reasons
peak_data_aligned <- align_chromatograms(data = peak_data, # input data
    rt_col_name = "time", # retention time variable name 
    rt_cutoff_low = 15, # remove peaks below 15 Minutes
    rt_cutoff_high = 45, # remove peaks exceeding 45 Minutes
    reference = NULL, # choose automatically 
    max_linear_shift = 0.05, # max. shift for linear corrections
    max_diff_peak2mean = 0.03, # max. distance of a peak to the mean across samples
    min_diff_peak2peak = 0.03, # min. expected distance between peaks 
    blanks = "C2", # negative control
    delete_single_peak = TRUE, # delete peaks that are present in just one sample 
    write_output = NULL) # add variable names to write aligned data to text files
```

The aligned data matrices are now stored in data frames which can be accessed as follows:

```
peak_data_aligned$aligned$time # to access the aligned retention times
peak_data_aligned$aligned$area # to access the aligned area data
```

The package includes the already aligned data set for all samples using default alignment parameter settings.

```
data("aligned_peak_data")
```

### Visual diagnostics for the aligned data

The `gc_heatmap` function can be used to visualise aligned datasets. A white filling indicates the absence of a peak in a sample, when using the default option of a binary heatmap. The basic rationale of the alignment is to sort the substances with very similar retention times together, as they represent putatively homologous substances. The heatmap shows how single peaks for the individuals deviate from the mean retention time of a substance. As a rule of thumb: The larger the deviation, the less likely it is the same substance, but retention time variation can be high for certain peaks. Therefore, retention time variations are highlighted with respect to mean and outliers are highlighted. By default, the a threshold value matching the parameter `max_diff_peak2peak` is used to show deviations. However, deviations might be higher after pairs of rows where merged with respect to the value of `min_diff_peak2peak`. Check out the documentation with `help("gc_heatmap")` for further possibilities.

```
gc_heatmap(aligned_peak_data,threshold = 0.03)
```

Heatmap showing the distribution of peaks and the variability within substances

The plot function shows a four figure plot for the aligned data. The first histogram shows the number of peaks per sample before and after alignment. The number of peaks is much smaller after the alignment as peaks have been deleted which were present in the control samples, as well as peaks that were found in just a single individual. The histograms on the bottom left shows the full chromatogram shifts (the first step in the algorithm). The bottom middle shows how much peaks vary around their means across samples. The histogram on the bottom right shows how many peaks are shared across samples. In this case, there is just a single substance present in all samples, while often 10-12 samples share a single substance (the mode of the distribution).

```
plot(aligned_peak_data,which_plot = "all") # Plots, can be invoked separetely
```

Diagnostic plot of the aligned dataset

Using the `print`function, provides a verbal summary of the alignment procedure is retrieved and the function call can be retraced.

```
print(aligned_peak_data) 
#> Summary of Peak Alignment running align_chromatograms
#> Input: peak_data
#> Start:  2017-07-19 16:31:47  Finished:  2017-07-19 17:30:47 
#> 
#> Call:
#>   GCalignR::align_chromatograms(data=peak_data, rt_col_name=time,
#>   max_linear_shift=0.05, max_diff_peak2mean=0.02, min_diff_peak2peak=0.08,
#>   blanks=(C2, C3), delete_single_peak=T, sep=\t, rt_cutoff_low=NULL,
#>   rt_cutoff_high=NULL, reference=NULL)
#> 
#> Summary of scored substances:
#>    total   blanks singular retained 
#>      494      171       45      278 
#> 
#> In total 494 substances were identified among all samples. 171 substances were
#>   present in blanks. The corresponding peaks as well as the blanks were removed
#>   from the data. 45 substances were present in just one single sample and were
#>   removed. 278 substances are retained after all filtering steps.
#> 
#> Sample overview:
#>   The following 84 samples were aligned to the reference 'P20':
#>   M2, M3, M4, M5, M6, M7, M8, M9, M10, M12, M14, M15, M16, M17, M18, M19, M20,
#>   M21, M23, M24, M25, M26, M27, M28, M29, M30, M31, M33, M35, M36, M37, M38, M39,
#>   M40, M41, M43, M44, M45, M46, M47, M48, P2, P3, P4, P5, P6, P7, P8, P9, P10,
#>   P12, P14, P15, P16, P17, P18, P19, P20, P21, P23, P24, P25, P26, P27, P28, P29,
#>   P30, P31, P33, P35, P36, P37, P38, P39, P40, P41, P43, P44, P45, P46, P47, P48
#> 
#> For further details type:
#>   'gc_heatmap(aligned_peak_data)' to retrieve heatmaps
#>   'plot(aligned_peak_data)' to retrieve further diagnostic plots
```

### Normalise peaks and log+1 transformation

`norm_peaks` is used to standardize the concentration of peaks across samples to obtain the relative abundance. This is an essential step prior to the analysis if the absolute concentration of chemicals varies across samples. Note that this step is also required when retention time cut-offs, single peak deletion or blank peak removal was applied, even if the data already contained a measure of relative abundance. The output is a list of data frames containing the relative abundance of peaks for every individual.

```
## normalise area and return a data frame
scent <- norm_peaks(aligned_peak_data, conc_col_name = "area",rt_col_name = "time",out = "data.frame") 
## common transformation for abundance data to reduce the extent of mean-variance trends
scent <- log(scent + 1)
```

### Visualise patterns by ordination plots using the *vegan* package

`vegan` offers a variety of useful function for the analysis of multivariate abundance data such as the scent profiles handled here. Check out `help("vegan")` for a first overview.

### Non-metric multidimensional scaling

```
## GCalignR contains factors for the chemical dataset
data("peak_factors") 
## keep order of rows consistent
scent <- scent[match(row.names(peak_factors),row.names(scent)),] 
## NMDS using Bray-Curtis dissimilarities
scent_nmds <- vegan::metaMDS(comm = scent, distance = "bray")
## get x and y coordinates
scent_nmds <- as.data.frame(scent_nmds[["points"]])  
## add the colony as a factor to each sample
scent_nmds <- cbind(scent_nmds,colony = peak_factors[["colony"]])
```

```
## ordiplot with ggplot2
library(ggplot2)
ggplot(data = scent_nmds,aes(MDS1,MDS2,color = colony)) +
    geom_point() + 
    theme_void() + 
    scale_color_manual(values = c("blue","red")) +
    theme(panel.background = element_rect(colour = "black", size = 1.25,
            fill = NA), aspect.ratio = 1, legend.position = "none")
```

NMDS plot

### Multivariate analysis using *adonis*

Using **adonis** and **betadisper** we can immediately do multivariate statistics, showing that the two colonies differ significantly. This illustrates a location effect.

```
## colony effect
vegan::adonis(scent ~ peak_factors$colony,permutations = 999) 
#> 
#> Call:
#> vegan::adonis(formula = scent ~ peak_factors$colony, permutations = 999) 
#> 
#> Permutation: free
#> Number of permutations: 999
#> 
#> Terms added sequentially (first to last)
#> 
#>                     Df SumsOfSqs MeanSqs F.Model     R2 Pr(>F)    
#> peak_factors$colony  1     2.543 2.54300  11.733 0.1279  0.001 ***
#> Residuals           80    17.340 0.21675         0.8721           
#> Total               81    19.883                 1.0000           
#> ---
#> Signif. codes:  0 '***' 0.001 '**' 0.01 '*' 0.05 '.' 0.1 ' ' 1
## no dispersion effect
anova(vegan::betadisper(vegan::vegdist(scent),peak_factors$colony))
#> Analysis of Variance Table
#> 
#> Response: Distances
#>           Df   Sum Sq   Mean Sq F value Pr(>F)
#> Groups     1 0.000791 0.0007913   0.222 0.6388
#> Residuals 80 0.285197 0.0035650
```

## References

Ottensmann, Meinolf, Martin A Stoffel, Hazel Nichols, and Joseph I Hoffman. 2017. “GCalignR: An R Package for Aligning Gas-Chromatography Data.” *bioRxiv*. Cold Spring Harbor Labs Journals. doi:10.1101/110494.

Stoffel, Martin A, Barbara A Caspers, Jaume Forcada, Athina Giannakara, Markus Baier, Luke Eberhart-Phillips, Caroline Müller, and Joseph I Hoffman. 2015. “Chemical Fingerprints Encode Mother–offspring Similarity, Colony Membership, Relatedness, and Genetic Quality in Fur Seals.” *Proceedings of the National Academy of Sciences* 112 (36). National Acad Sciences: E5005–E5012.
