# Supplementary material for: GCalignR: An R package for aligning gas-chromatography data for ecological and evolutionary studies
Source: PLoS One. 2018 Jun 7;13(6):e0198311. doi: 10.1371/journal.pone.0198311 (PMC5991698; doi:10.1371/journal.pone.0198311)
Supplement: S5 File — (HTML) [file pone.0198311.s005.html]

GCalignR: How does the Algorithm work?


# GCalignR: How does the Algorithm work?

#### *Meinolf Ottensmann, Martin A. Stoffel, Hazel J. Nichols and Joseph I. Hoffman*

## Introduction

The intention of this vignette is to illustrate how the algorithms in `GCalignR` handles the data during each of the processing steps. Simultaneously we deploy some simple datasets that can be generated within R to visualise the outcome of the processing steps in an easy way. For further descriptions of the algorithm we refer to our manuscript (Ottensmann et al. 2017). Here, we give a detailed introduction into the concept behind the package and illustrate how it works by simulating simple datasets with arbitrary peaks. To enhance readability, not all code lines are show within this vignette, but they can be easily accessed by typing `browseVignettes("GCalignR")` and clicking on `R code`. The datasets are pseudo-randomly created and can therefore differ from run to run. For consistency, we supply the dataset that is used to demonstrate the alignment progress with the package. An accompanying vignette *“GCalignR: Step by step”* focuses on the workflow with this package and the integration into a broader analysis pipeline.

## What´s a Peak List and how to create one ?

`GCalignR` performs all steps on a so called peak list. Such a list can be generated for every chromatogram, defined as the output of a Chromatograph (e.g. **G**as **C**hromatography **F**lame **I**onization **D**etector, GC-FID) that plots the measured electric current over the time course of a separation run. **Figure 1** shows a chromatogram containing five peaks that represent five chemicals detected in this simulated sample. Here, all the distinct peaks approximate perfect Gaussian distributions and are clearly separated. Essentially every vendor of a Chromatograph offers software that allows to detect peaks (i.e. substances) in the generated signal based on procedures that take into account the shape of a peak, the noise level of the signal and many things more that are out of the scope of `GCalignR` and we refer to specialised resources (Grob and Kaiser 2004). Nevertheless, the quality of the chromatograms as well as a sophisticated way to detect and quantify peaks is a crucial step before one should start to think about aligning peaks for downstream analysis!

Figure 1. A Chromatogram plots an intensity signal over the course of a separation run.

In this example it would be adequate to define each peak by two values, the retention time and the peak height as a measure of the concentration, simply by calculating the local maxima. **Figure 2** shows the peaks annotated by the intensity. The dashed vertical lines indicate the retention time of the peaks maximum that is written on top of each of the five peaks.

Figure 2. Chromatogram with integrated peaks

Now these and further information (e.g. the peak area) can be summarised in the form of a peak list that contains information for every detected peak. One row of such a peak list refers to a single peak. In general, peaks are ordered with increasing retention time, thereby starting with the most volatile substances.

|  | time | height |
| --- | --- | --- |
| Peak 1 | 5.01 | 1.53 |
| Peak 2 | 10.02 | 1.11 |
| Peak 3 | 13.10 | 1.42 |
| Peak 4 | 20.22 | 1.05 |
| Peak 5 | 24.57 | 1.02 |

**Figure 3** shows chromatograms of four samples “A1” to “A4” that were analysed on the same hypothetical GC-FID run. These peaks can be individually characterised by their retention times (see labels on each peak). In this small set of samples, one can easily see that several peaks appear in consistent temporal sequence with increasing retention times in sample order approximating intervals of 0.7 minutes. Here, it would be possible to account for this variation manually, but consider a scenario where there are many more samples and peaks, perhaps in noisier chromatograms. For this reason we developed `GCalignR` and implemented simple algorithms that are explained below. Before these chromatograms can be analysed, we need to obtain peak retention times and peak heights again. Additionally we need to format it for using `GCalignR` to align the peaks. `GCalignR` is distributed with sample files that can be used as templates.

Figure 3. Overlay of Chromatograms from four samples

The standard input format is a tab-delimited text file that contains all the required information.(1) The first row lists the sample identifiers of all the samples included for aligning, (2) column names are written in the second row and specify the content of individual peak lists that are (3) incorporated for each individual below. Peak lists have to be concatenated column-wise in the order specified in the first row. The input file belonging to the chromatograms shown in **Figure 3** is depicted below. Several sample files are distributed with `GCalignR`. Type `system.file("extdata", package = "GCalignR")` to obtain the path on your computer. Any row of a given sample that only contains zeros or NAs is treated as empty an will be deleted prior to running the algorithms.

```
> A1    A2  A3  A4
> 
> rt    height
> 1.7   1   2.6 1.42    3.1 1.05    3.6 1.25
> 9.7   1   10.4    1.42    11.1    1.66    12  1.99
> 13.7  1.21    14.2    1.53    15.1    1.48    15.6    1.48
> 17.9  1.17    18.4    1.81    19.1    1.81    20  1.38
> 21.7  1.21    22.4    1.48    23.3    1.14    23.8    1.66
> 25.7  1.05    26.6    1.66    0   0   28  1.48
```

## Aligning peak lists

Over the course of the analytic pipeline, retention times of the same substance can vary for a number of reasons that include column ageing, perturbations of the carrier gas flow or temperature fluctuations, all of which can be avoided with varying success. `GCalignR` comes into place, when a question regarding the similarity of a number of samples is addressed by analysing their chemical composition. For this purpose it is crucial to cluster peaks that belong to the putatively homologous substance across samples. All alignment steps take only the retention time of a peak into account and are embedded within a single function `align_chromatograms` that conducts the alignment sequentially. Any other variable included in the dataset remains always associated with the retention time of the peak and is not treated any further. The single steps undertaken by the function to align the data can be traced back from the output that is returned after execution of `align_chromatograms`.

### Full alignment of peaks lists

The first step in the alignment acts on linear drifts across samples. Therefore, a reference sample is required that will be selected automatically, by picking the sample with the highest average similarity to all others samples based on the original retention times. Otherwise, a reference can be selected manually. Linear shifts between each sample and the reference are evaluated in a pairwise comparison similar to a cross-correlation based on the peak retention time alone. Thereby, the sample is slided in a user-defined window in discrete time steps. **Figure 4** illustrates this for two samples (Sample “B” & “C”), where the linear shift was evaluated by in a window ranging from -2 to +2 minutes. Here, the “true” linear shift between reference (Sample A) and each sample is approximated by searching for the minimum total deviation in retention times between all reference peaks and the closest peak in a sample. When more than one shift size (including 0!) yield to the same similarity score, the smallest value is taken to avoid overshooting. In this example, Sample B is shifted by - 2 and Sample C by + 1, whereas all other considered steps would result in greater deviations in retention times to the reference peaks. However, it is important to note that putatively three peaks are shared between the reference Sample A and Sample B and Sample C respectively, but due to non-linear perturbations not all peaks align well after the full alignment took place.

Figure 4. Corrected linear drift between Reference and Focal sample at a shift of -1

### Partial alignment of peaks

As shown in **Figure 4**, non-linear errors in retention times need to be accounted for in order to cluster similar retention times that belong to putatively homologous substances. This step of the algorithm takes the linearly adjusted peak retention times as input, but does not manipulate them any further. All computation is from now onwards based on the concept of a retention matrix, in which columns represent samples and rows are the operational unit of a substance. Because of the more or less subtle variations in the retention times, the core alignment is focusing one substance (i.e. row of the matrix) at a time and works itself through all the substances it will get to see, starting with the first row in the matrix. **Figure 5** shows the matrix operations for an already linearly corrected example. All the operations are based on the comparison of a focal retention time with the mean retention time of all previous samples within the same row, always starting with the first column. If a focal retention time deviates significantly from the mean a matrix manipulation is conducted to solve the conflict. Whenever the focal peak has a larger retention time, a gap is introduced by inserting a zero and moving the peak to the next row, whereas a gap is introduced in all previous samples is introduced if the focal peak has a smaller retention time value. Here, a user-defined threshold, set by the parameter `max_diff_peak2mean = 0.5` in this example, defines when manipulations will be conducted. In order to follow the steps more easily, the order of samples is kept constant in this illustration, whereas the implemented algorithm uses random starts for each row to prevent any systematic errors that remained until now, from inflating results.

Figure 5. Alignment of individual peaks based on retention time matrices. Colours represent substances, black rectangles highlight causes of manipulations.

## Merging rows

Looking at final retention matrix (bottom left) in Figure 5, we immediately see that rows 1 and 2 have very similar mean retention times. Here, the difference is even smaller than specified by `max_diff_peak2mean` and the separation was triggered *accidentally* by comparing the two extreme values prior to the first manipulation (upper left matrix). For this and similar situations, a third step in the alignment is available that tries to merge rows that differ by (i) less than `min_diff_peak2peak` in mean retention times and (ii) show a clear pattern, by which not a single sample shows peaks in all of the focal rows. Setting `min_diff_peak2peak` to a value of two-times `max_diff_peak2mean` will solve this conflict by merging both of the rows (Figure 6).

Figure 6. Merging redudant rows of homologous peaks.

## Aligning the a hypothetical dataset

Now we are going to apply the alignment to a dataset that is highly similar to the example depicted in Figure 3. Here, all peaks are shown with the same shape, which is appropriate as we are only interested in the retention times. Based on the inspection of the graph shown in **Figure 7**, we pick “A2” as a reference and take into account that peaks of “A3” and “A4” are seemingly postponed by approx. 0.7 and 1.4 minutes, whereas “A1” shows peaks 0.7 minutes earlier. Therefore, a good estimate for the required window size to correct for linear shifts is given by `max_linear_shift = 1.6` yielding to a window of 1.6 Minutes around the retention times of the reference sample, including a safety margin. We can check if the search window is of appropriate size after executing the algorithm. For aligning individual peaks were stick to the default values `max_diff_peak2mean = 0.02` and `min_diff_peak2peak = 0.08` for now.

```
## path to the data
path <- system.file("extdata", "simulated_peak_data.txt", package = "GCalignR")
## draw chromatograms
x <- draw_chromatogram(data = path, rt_col_name = "rt", show_rt = T, show_num = F, plot = F)
x[["ggplot"]] + geom_line(size = 1.2) + theme(axis.ticks.x = element_blank()) + ggplot2::scale_color_brewer(palette = "Dark2")
```

Figure 7. Chromatographic representation of the dataset prior to alignment

```
path <- system.file("extdata", "simulated_peak_data.txt", package = "GCalignR")
aligned <- align_chromatograms(data = path,
                               rt_col_name = "rt",
                               max_linear_shift = 1.6,
                               max_diff_peak2mean = 0.5,
                               min_diff_peak2peak = 1,
                               reference = "A2")
```

`GCalignR` creates a *Logfile* while processing a dataset that allows to trace back the Linear shifts that have been applied to the samples. We can see that the linear shifts of “A3” and “A4” are of the size that we expected, whereas the drift in “A1” was putatively not fully compensated with 0.5 instead of 0.7. The maximum value is -1.4 and therefore the setting `max_linear_shift = 1.6` was enough.

```
print(aligned[["Logfile"]][["LinearShift"]])
>   shift sample
> 1   0.7     A1
> 2   0.0     A2
> 3  -0.7     A3
> 4  -1.4     A4
```

For a bigger dataset it is more convenient to invoke a histogram that shows the distribution of applied shifts (**Figure 8**). Here, the horizontal axis shows the range that was considered. As a rule of thumb, a skew towards one or the other end of the axis would indicate a potential underestimation of the drift amplitude.

```
plot(aligned, which_plot = "shifts")
```

Figure 8. Histogram of linear shifts.

Using `draw_chromatograms` again, we can inspect how the linear corrections have changed the peak list.

```
x <- draw_chromatogram(data = aligned, rt_col_name = "rt", step = "shifted", show_rt = F, show_num = F, plot = F)
x[["ggplot"]] + ggplot2::scale_color_brewer(palette = "Dark2")
```

Figure 9. Correcting linear drift reduces the gaps between homologous peak retention times

We immediately see that the peaks were shifted accordingly and start to cluster as expected (**Figure 9**). However, there is variation in retention times among the samples within the visually separated clusters. Therefore, we utilise another algorithm that evaluates the observed variance and decides which peaks belong to the same substance. These steps were already executed with the call to `align_chromatograms` and we can inspect the results by simply defining `step = "fully_aligned"`. This time we also set `show_num = T` in order to print the number of samples *behind* each peak. This is helpful, because peaks of the same substance will overlap indicating that the retention time is exactly matched (**Figure 10**).

```
x <- draw_chromatogram(data = aligned, rt_col_name = "rt", step = "aligned", show_num = T, plot = F)
x[["ggplot"]] + ggplot2::scale_color_brewer(palette = "Dark2")
```

Figure 10. Aligned peaks are represented by the mean retention time of a substance

We can test that not only “A4” contributes to the peaks by moving each sample to its own panel on the plot (**Figure 11**) by making use of the `facet_wrap` function in `ggplot2`. The data frame that is used to create the plot is accessible in the list that is returned by a call to `draw_chromatogram`.

```
## for using ggplot2::facet_wrap we need to get rid of the annotations
x <- draw_chromatogram(data = aligned, rt_col_name = "rt", step = "aligned", show_num = F, plot = F)
> Computing chromatograms ...
x[["ggplot"]] + ggplot2::facet_wrap(~sample, ncol = 1) + ggplot2::scale_color_brewer(palette = "Dark2")
```

Figure 11. Suplots are a convenient means of visualising aligned peak lists

## Remarks

Running `align_chromatograms` with default settings will be a good starting point for aligning a dataset and we were able to show that parameter settings are generally robust (Ottensmann et al. 2017). However, every dataset has unique features that will require to change one or more of the two parameters `max_diff_peak2mean` and `min_diff_peak2peak`. For the example above, we created a dataset by picking peaks pseudo-randomly and adding arbitrary perturbations. Especially the amplitude of linear drift in the range of minutes is not expected in *real life* applications of chromatography and was used to illustrate the principles. When one works with experimental data we suggest to use the original chromatograms in combination with the `draw_chromatogram` tool to explore the data carefully, for example by looking at subsets of samples and different time scales. All of the optional parameters that enable options of filtering and preprocessing need to be applied with caution. For example, excluding peaks that are unique for a single sample is adequate for similarity analyses but not helpful for characterising the composition of a sample and is also possible afterwards. In another vignette `GCalignR step by step` we focus on a empirical dataset and illustrate how `GCalignR` can be used within a broader workflow.

## References

Grob, Robert L., and Mary A. Kaiser. 2004. “Qualitative and Quantitative Analysis by Gas Chromatography.” In *Modern Practice of Gas Chromatography*, 403–60. John Wiley & Sons, Inc. doi:10.1002/0471651141.ch8.

Ottensmann, Meinolf, Martin A Stoffel, Hazel Nichols, and Joseph I Hoffman. 2017. “GCalignR: An R Package for Aligning Gas-Chromatography Data.” *bioRxiv*. Cold Spring Harbor Labs Journals. doi:10.1101/110494.
